# A Comprehensive Landscape of *De Novo* Malignancy After Double Lung Transplantation

**DOI:** 10.3389/ti.2023.11552

**Published:** 2023-08-17

**Authors:** Jeeyeon Lee, Andrew Won Jun Yang, Liam Il-Young Chung, Jisang Yu, Yunjoo Lee, Hye Sung Kim, Hyun Joon Shin, Young-Geun Choi, Ankit Bharat, Young Kwang Chae

**Affiliations:** ^1^ Department of Surgery, School of Medicine, Kyungpook National University Chilgok Hospital, Kyungpook National University, Daegu, Republic of Korea; ^2^ Department of Internal Medicine, Northwestern Memorial Hospital, Chicago, IL, United States; ^3^ Division of Cardiology, Department of Medicine, Lemuel Shattuck Hospital, Massachusetts Department of Public Health, Jamaica Plain, MA, United States; ^4^ Department of Mathematics Education, Sungkyunkwan University, Seoul, Republic of Korea

**Keywords:** post-transplant malignancy, *de novo* malignancy, double lung transplant, incidence, survival outcomes

## Abstract

Although the association between post-transplant malignancy (PTM) and immunosuppressive therapy after organ transplantation has been studied, an integrated review of PTM after lung transplantation is lacking. We investigated the incidence and types of *de novo* PTM and its impact on survival following double lung transplantation (DLT). The incidence and type of PTM as well as the annual and cumulative risks of each malignancy after DLT were analyzed. The overall survival (OS) of recipients with or without PTM was compared by the Kaplan–Meier survival method and landmark analysis. There were 5,629 cases (23.52%) with 27 types of PTMs and incidences and OS varied according to the types of PTMs. The recipients with PTM showed a significantly longer OS than those without PTM (*p* < 0.001). However, while the recipients with PTM showed significantly better OS at 3, and 5 years (*p* < 0.001, *p* = 0.007), it was worse at the 10-year landmark time (*p* = 0.013). And the single PTM group showed a worse OS rate than the multiple PTM group (*p* < 0.001). This comprehensive report on PTM following DLT can help understand the risks and timing of PTM to improve the implementation of screening and treatment.

## Graphical Abstract

**Figure:**
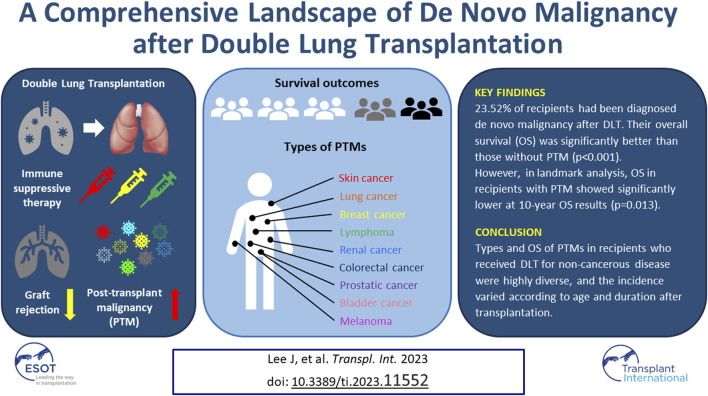

## Introduction

Over the past 20 years, there has been a notable increase in thoracic organ transplantation, with double lung transplantation (DLT) surpassing single lung transplantation nearly two-fold since 2005 [1–4]. Immunosuppressive therapy has substantially improved post-transplant outcomes by mitigating acute and chronic rejection episodes [5–7]. The standard immunosuppressive regimen for lung transplantation consists of calcineurin inhibitors, antimetabolites, and corticosteroids [8, 9]. This regimen has effectively reduced allograft tissue rejection and graft failure, enhancing transplant recipients’ survival outcomes [10, 11].

The immunosuppressive regimen attenuates the signaling between antigen-presenting cells and T-cells, inhibits T-cell activation and proliferation, reduces antibody production by B cells, and suppresses antibody-mediated complement system activation [12–14]. However, this immunosuppressive microenvironment may inadvertently promote tumor development and progression, facilitating immune evasion by cancer cells [15, 16]. Consequently, while immunosuppressive therapy has successfully suppressed allograft rejection, malignancies associated with immunosuppression are increasingly acknowledged as a significant post-transplant complication [17, 18].

Although the relationship between post-transplant malignancy (PTM) and immunosuppressive therapy has been suggested, PTM remains a leading cause of mortality in thoracic transplantation patients [19–21]. Transplant recipients face a lifelong risk of PTM, necessitating diligent screening for *de novo* PTM. A thorough examination of PTM, accounting for transplant recipient characteristics and time since transplantation, is crucial for informing PTM management strategies.

In this study, we investigated the annual incidence, cumulative risk, and survival outcomes of PTM in patients who underwent DLT for non-cancerous diseases. We utilized data from the Organ Procurement and Transplantation Network (OPTN) to better understand PTM characteristics following DLT.

## Material and Methods

Data: Data pertaining to thoracic transplantation was procured from the United Network for Organ Sharing (UNOS)—a non-profit organization committed to its mission of overseeing the nation’s transplant system under the purview of the federal government^1^. The data, which were de-identified, anonymized, and accompanied by coding files in STATA format, were sourced from the thoracic transplant registry of the OPTN as of 7 October 2022. Only DLT recipients were included while single or multi-organ transplants were excluded given the potential for confounding bias. Among the 29,335 documented DLT cases conducted between 1993 and June 2022, a total of 23,935 recipients who had eligible data were ultimately assessed for *de novo* PTMs following DLT, upon reviewing data suitability. Recipients who had undergone DLT for malignancy were excluded from the study, which received approval from Northwestern University’s Institutional Review Board Committee in Chicago, IL, United States (IRB#: STU00207117). The collected data encompassed recipient age at the time of transplantation, sex, smoking history, prior indication for DLT, presence, and date of *de novo* PTM, PTM type, date and cause of death. The recipient cohorts were divided into two groups: those without *de novo* PTM (*n* = 18,306) and those with *de novo* PTM (*n* = 5,629). The incidence, annual and cumulative risks of each PTM subtype were scrutinized, and survival outcomes were contrasted.

Analysis: Clinical factors and survival outcomes were evaluated at 5 and 10 years post-DLT for all recipients. Incidence, as well as annual and cumulative risks of PTM, were computed according to PTM type. Furthermore, the variation in annual risk proportion was compared as the follow-up period extended. With a follow-up period of at least 18 years, the cumulative risk was ascertained for the four most prevalent PTM causes: squamous cell skin cancer (SCC), basal cell skin cancer (BCC), lymphoma, and lung cancer. Recipients with PTM were further categorized based on the number of PTMs they developed, and the overall survival (OS) was analyzed for statistical differences based on the number of PTMs.

Statistics: Quantitative variables were compared using the *t*-test, and categorical variables were analyzed using the χ^2^ test. The survival outcomes were analyzed with the Kaplan–Meier survival method. For multivariate analysis, the Cox regression analysis was performed, considering age, sex, and cigarette use at the time of DLT as the variables. For the landmark analysis, we chose 3, 5, 7, 10, 15, and 20 years after transplantation as landmark time points. Only patients alive at this point were included in this analysis and performed an analysis with recipients with or without PTM before time points. All statistical analyses were performed using the SPSS software (version 29.0 SPSS, IBM, Chicago, IL, United States), and a *p*-value of <0.05 was used to determine statistical significance.

## Results

### Clinical and Demographic Features

Among the 23,935 DLT recipients, 13,768 (57.52%) were males, and 11,129 (46.50%) had a smoking history. The mean age of the recipients was 51.91 years (SD, ±4.95). During the follow-up period, 5,629 cases (30.75%) of PTM occurred, and the mean age of recipients with PTM was significantly greater than that of those without PTM [without PTM: 51.13 years (SD, ±41.72) versus those with PTM: 54.46 years (SD, ±19.09), *p* < 0.001]. Male DLT recipients (*n* = 3,785, 67.24%) were more frequently diagnosed with PTMs (*p* < 0.001), and the mean age at the onset of PTMs was 60.87 years (SD, ±49.26) (Table 1).

**TABLE 1:** Characteristics of recipients with or without *de novo* post-transplant malignancy (PTM) who had received double lung transplantation for non-cancerous diseases.

Variables	Total (*n* = 23,935)	Recipients without PTM (*n* = 18,306)	Recipients with PTM (*n* = 5,629)	*p*-value*
Age at transplantation (mean, ±SD)	51.91 ± 4.95	51.13 ± 41.72	54.46 ± 19.09	<0.001
Gender (*n*, %)				<0.001
Male	13,768 (57.52)	9,983 (54.53)	3,785 (67.24)	
Female	10,167 (42.48)	8,323 (45.47)	1,844 (32.76)	
Smoking history (*n*, %)				<0.001
Non-smoker	9,148 (38.22)	7,490 (40.92)	1,658 (29.45)	
Smoker	11,129 (46.50)	8,282 (45.24)	2,847 (50.58)	
Unknown	3,658 (15.28)	2,534 (13.84)	1,124 (19.97)	
Death (*n*, %)				<0.001
No	12,216 (51.04)	9,794 (53.50)	2,421 (43.01)	
Yes	11,719 (48.96)	8,512 (46.50)	3,208 (56.99)	
Onset of PTM from transplantation				N/A
Median (months, range)	—	—	47.97 (0.00–316.10)	
Mean (months, ±SD)	—	—	60.87 ± 49.26	

* Quantitative variables were compared using a *t*-test, and categorical variables were analyzed using the χ^2^ test.

### Indications of DLT for Non-Cancerous Disease

There were 87 different indications for DLT, with the most common being idiopathic pulmonary fibrosis/usual interstitial pneumonitis (*n* = 6,400; 23.74%). The second and third most common indications for DLT were chronic obstructive pulmonary disease/emphysema (*n* = 5,276; 22.04%), and cystic fibrosis (*n* = 4,075; 17.03%). The order of common indications for DLT was identical in recipients with and without PTM (Supplementary Table S1).

### Types and Incidences of *De Novo* PTM

**FIGURE 1:**
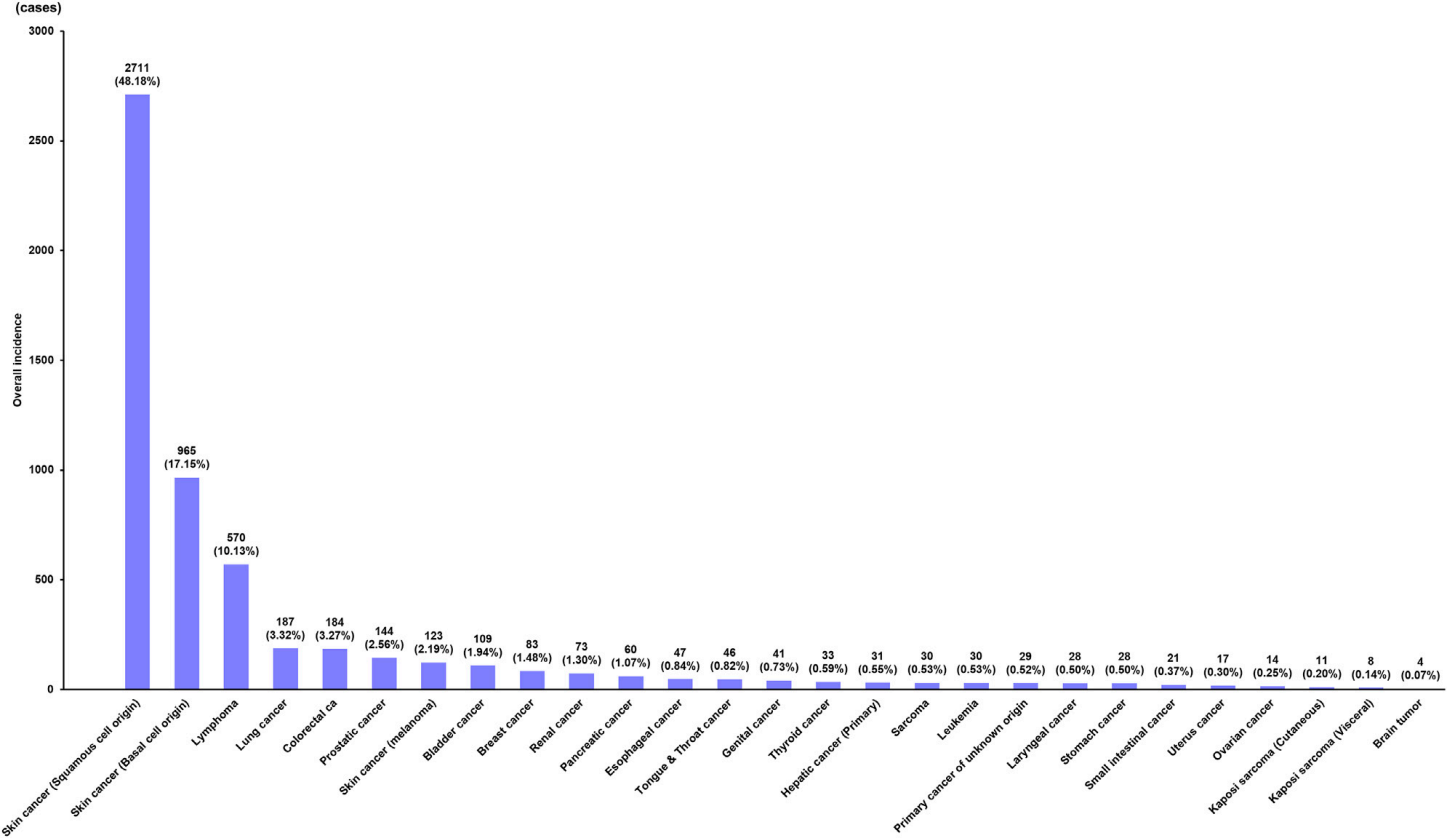
Incidence of *de novo* post-transplant malignancy after double lung transplantation.

Twenty-seven types of *de novo* PTM were detected after DLT for non-cancerous disease during the surveillance. The common tumor types were SCC (*n* = 2,711; 48.16%), BCC (*n* = 965; 17.14%), lymphoma (*n* = 570; 10.13%), lung cancer (*n* = 187; 3.32%), colorectal cancer (*n* = 184; 3.27%), prostatic cancer (*n* = 144; 2.56%), skin cancer (melanoma) (*n* = 123; 2.19%), bladder cancer (*n* = 109; 1.94%), breast cancer (*n* = 83; 1.47%), renal cancer (*n* = 73; 1.30%), pancreatic cancer (*n* = 60; 1.07%), esophageal cancer (*n* = 47; 0.83%), tongue and throat cancer (*n* = 46; 0.82%), genital cancer including vulva, peritoneum, penis, and scrotum (*n* = 41; 0.73%), thyroid cancer (*n* = 33; 0.59%), primary hepatic cancer (*n* = 31; 0.55%), sarcoma (*n* = 30; 0.53%), leukemia (*n* = 30; 0.53%), primary cancer of unknown origin (*n* = 29; 0.52%), stomach cancer (*n* = 28; 0.50%), laryngeal cancer (*n* = 28, 0.50%), small intestinal cancer (*n* = 21; 0.37%), uterus cancer (*n* = 17; 0.30%), ovarian cancer (*n* = 14; 0.25%), Kaposi sarcoma (cutaneous type) (*n* = 11; 0.20%), Kaposi sarcoma (visceral type) (*n* = 8; 0.14%), and brain tumor (*n* = 4; 0.07%) (Figure 1).

### Annual Risks of Each *De Novo* PTM After DLT

**FIGURE 2:**
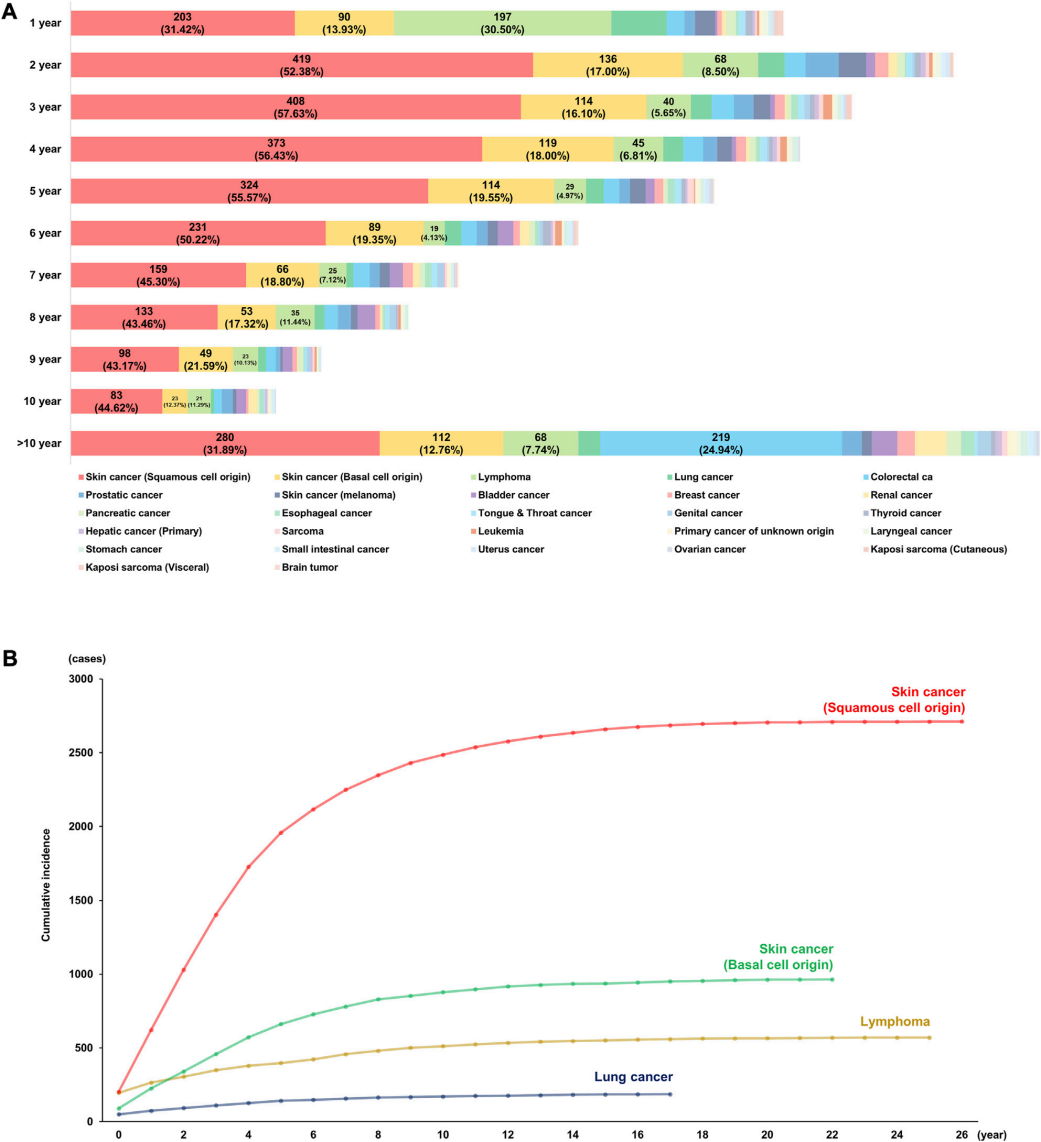
Incidence of *de novo* malignancy after double lung transplantation. **(A)** Annual incidences of post-transplant malignancy. **(B)** Cumulative risks of top four causes of post-transplant malignancy.

The lifetime incidence of *de novo* PTM following DLT was identified as 23.52% (5,629/23,935), and the annual risks of *de novo* malignancy after DLT are shown in Figure 2A. During the first year following DLT, SCC (*n* = 203; 31.42%) occurred most frequently, followed by lymphoma (*n* = 197; 30.50%), BCC (*n* = 90; 13.93%), and lung cancer (*n* = 50; 7.74%). SCC was diagnosed more than twice as frequently in the first year and most frequently in the second year following DLT (*n* = 419; 52.38%), and then gradually decreased. Although BCC also occurred more frequently in the second year than in the first year following DLT, the range of change was smaller than that of SCC. Lymphoma and lung cancer most frequently occurred during the first year of DLT; the incidence decreased to less than half in the second year following DLT and gradually decreased thereafter. Although the incidence of colorectal cancer was less than 20 per year during the first 10 years, they continued to occur even 10 years after DLT.

### Cumulative Risks of Each *De Novo* PTM After DLT

The incidence of SCC increased until 10 years and rarely occurred 20 years following DLT, and the total cumulative incidence was 2,711 (48.16%). On the other hand, BCC was the second most common PTM after DLT and the rate of increase was slower than that of SCC. Lymphoma was the third most common PTM with a cumulative incidence of 570 (10.13%), and one-third of cases occurred during the first year of transplantation (*n* = 197/570, 34.56%). Lung cancer was the fourth most common PTM with a cumulative incidence of 119 (3.55%) during the 18 years of follow-up.

The cumulative risks of PTM after DLT are shown in Figure 2B (the top four causes: SCC, BCC, lymphoma, and lung cancer) and Supplementary Figure S1 (the other causes of PTM).

### Age Distribution of Recipients With *De Novo* PTM After DLT

**FIGURE 3:**
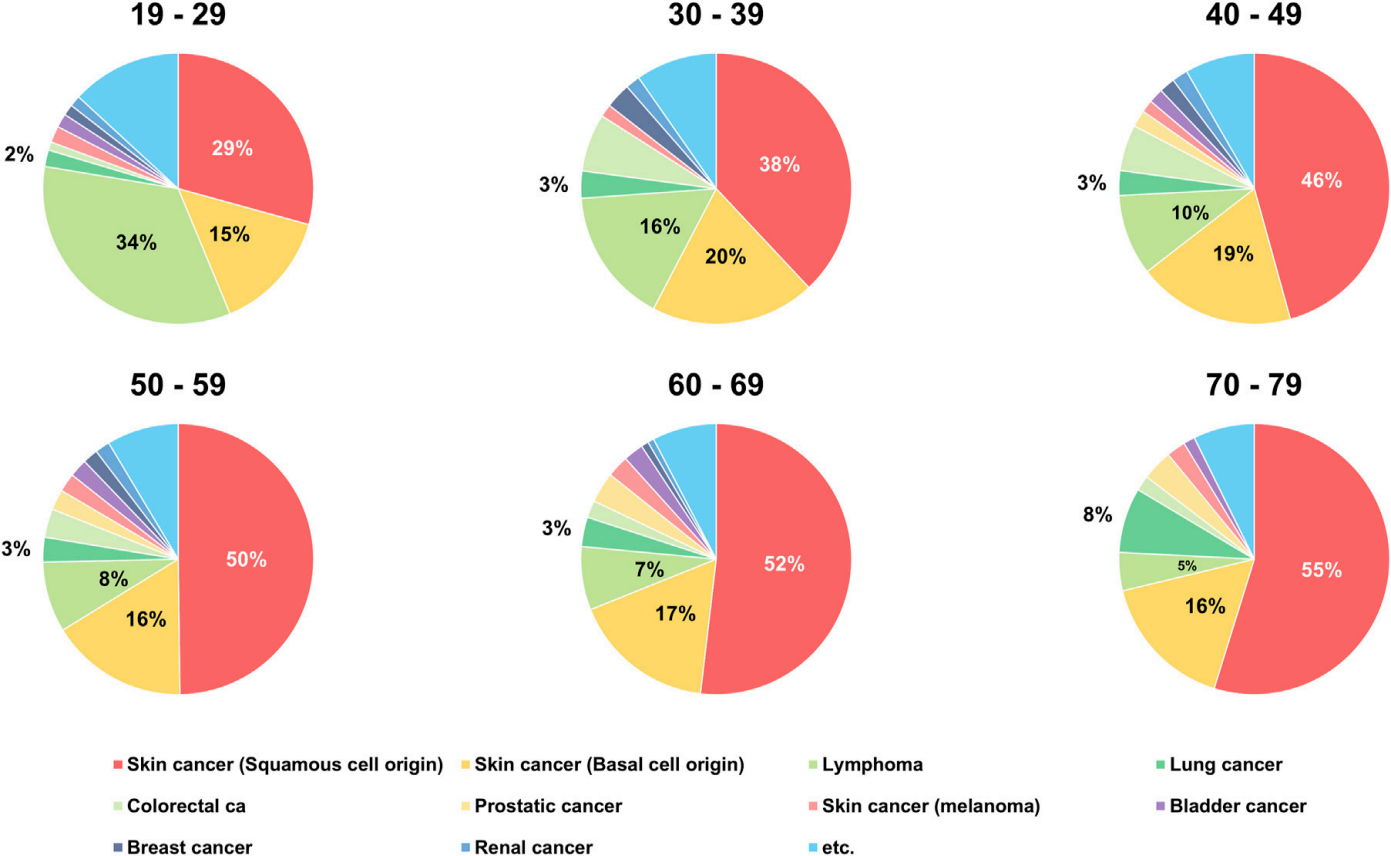
Age distribution of recipients with *de novo* post-transplant malignancy after double lung transplantation.

When the incidence of PTMs was analyzed by age group, SCC was the most common PTM in all age groups except for recipients aged 19–29 years. In recipients aged 19–29 years, lymphoma (*n* = 103, 33.88%) was the most common tumor type after DLT. While the incidence of lymphoma gradually decreased with age, BCC showed similar rates of incidence in all age groups (range, 14.47%–19.65%). The incidence of lung cancer after DLT showed similar rates among recipients in the 19–69 age group except for those in the 70–79 age group (*n* = 17, 7.76%). The incidence of colorectal cancer after DLT was higher in recipients aged 30–39 and 40–49 years than in other age groups (Table 2; Figure 3).

**TABLE 2:** Sex and age distributions in recipients with post-transplant malignancy (PTM) after double lung transplantation.

Variables (*n*, %)	Sex	Age groups					
	Male: Female	19–29	30–39	40–49	50–59	60–69	70–79
Total recipients	13,768 (57.52): 10,167 (42.48)	2,466	2,457	3,312	7,005	7,842	853
Recipients with PTM	3,785 (67.24): 1,844 (32.76)	304 (12.33)	463 (18.84)	744 (22.46)	1,759 (25.11)	2,140 (27.29)	219 (25.67)
Type of PTMs							
Skin cancer (Squamous cell origin)	1,942 (71.63): 769(28.37)	89 (29.28)	176 (38.01)	340 (45.7)	876 (49.8)	1,110 (51.87)	120 (54.79)
Skin cancer (Basal cell origin)	655 (67.88): 310 (32.12)	44 (14.47)	91 (19.65)	140 (18.82)	289 (16.43)	365 (17.06)	36 (16.44)
Lymphoma	336 (58.95): 234 (41.05)	103 (33.88)	75 (16.20)	72 (9.68)	148 (8.41)	162 (7.57)	10 (4.57)
Lung cancer	127 (67.91): 60 (32.09)	6 (1.97)	15 (3.24)	22 (2.96)	52 (2.96)	75 (3.50)	17 (7.76)
Colorectal ca	94 (51.09): 90 (48.91)	3 (0.99)	32 (6.91)	41 (5.51)	60 (3.41)	44 (2.06)	4 (1.83)
Prostatic cancer	144 (100.00): 0 (0.00)	0 (0.00)	0 (0.00)	15 (2.02)	43 (2.44)	78 (3.64)	8 (3.65)
Skin cancer (melanoma)	82 (66.13): 42 (33.87)	6 (1.97)	7 (1.51)	11 (1.48)	39 (2.22)	56 (2.62)	5 (2.28)
Bladder cancer	81 (73.64): 29 (26.36)	5 (1.64)	0 (0.00)	13 (1.75)	38 (2.16)	51 (2.38)	3 (1.37)
Breast cancer	2 (2.41): 81 (97.59)	4 (1.32)	14 (3.02)	14 (1.88)	32 (1.82)	19 (0.89)	0 (0.00)
Renal cancer	56 (76.71): 17 (23.29)	4 (1.32)	8 (1.73)	14 (1.88)	31 (1.76)	16 (0.75)	0 (0.00)
Others	266 (55.65): 212 (44.35)	40 (13.16)	45 (9.72)	62 (8.33)	151 (8.58)	164 (7.66)	16 (7.31)

### Incidence of *De Novo* PTM by Order of Occurrence in Recipients With Multiple PTM

**FIGURE 4:**
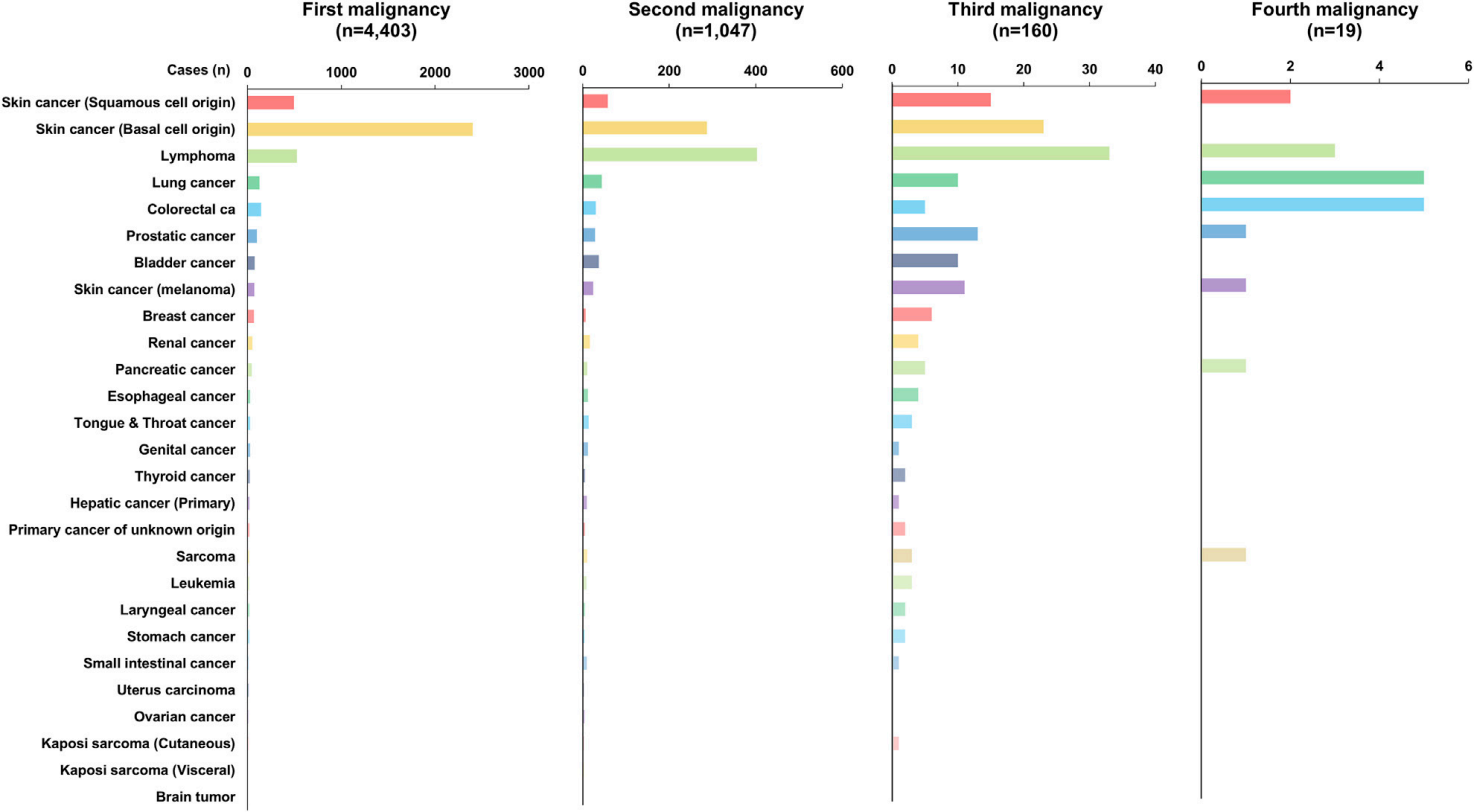
Incidence of *de novo* PTM depending on the order of occurrence in recipients with PTM.

A total of 4,403 recipients (78.22%) were diagnosed with a single *de novo* PTM after DLT, and 1,047 recipients (18.60%) were diagnosed with double *de novo* PTMs simultaneously or subsequently. Furthermore, 160 recipients (2.84%) and 19 recipients (0.34%) were diagnosed with three and four *de novo* PTMs, respectively. While BCC was the most common tumor type (*n* = 2,401; 54.53%) in the first malignancy group, lymphoma was the most common tumor type in the second (*n* = 403, 38.49%), and third (*n* = 33, 20.63%) malignancy groups. Brain tumors (n = 4, 0.09%) occurred only in recipients who had a single PTM (Table 3; Figure 4).

**TABLE 3:** Incidence of *de novo* malignancy after double lung transplantation by order of occurrence in recipients with single or multiple post-transplant malignancy (PTM).

Types of PTM (*n*, %)	Orders of *de novo* PTM			
	First malignancy (*n* = 4,403)	Second malignancy (*n* = 1,047)	Third malignancy (*n* = 160)	Fourth malignancy (*n* = 19)
Skin cancer (Squamous cell origin)	495 (11.24)	58 (5.54)	15 (9.38)	2 (10.53)
Skin cancer (Basal cell origin)	2,401 (54.53)	287 (27.41)	23 (14.38)	0 (0.00)
Lymphoma	526 (11.95)	403 (38.49)	33 (20.63)	3 (15.79)
Lung cancer	128 (2.91)	44 (4.20)	10 (6.25)	5 (26.32)
Colorectal ca	144 (3.27)	30 (2.87)	5 (3.13)	5 (26.32)
Prostatic cancer	102 (2.32)	28 (2.67)	13 (8.13)	1 (5.26)
Bladder cancer	77 (1.75)	37 (3.53)	10 (6.25)	0 (0.00)
Skin cancer (melanoma)	74 (1.68)	24 (2.29)	11 (6.88)	1 (5.26)
Breast cancer	70 (1.59)	7 (0.67)	6 (3.75)	0 (0.00)
Renal cancer	53 (1.20)	16 (1.53)	4 (2.50)	0 (0.00)
Pancreatic cancer	44 (1.00)	10 (0.96)	5 (3.13)	1 (5.26)
Esophageal cancer	31 (0.70)	12 (1.15)	4 (2.50)	0 (0.00)
Tongue and Throat cancer	29 (0.66)	14 (1.34)	3 (1.88)	0 (0.00)
Genital cancer	28 (0.64)	12 (1.15)	1 (0.63)	0 (0.00)
Thyroid cancer	26 (0.59)	5 (0.48)	2 (1.25)	0 (0.00)
Hepatic cancer (Primary)	21 (0.48)	9 (0.86)	1 (0.63)	0 (0.00)
Primary cancer of unknown origin	23 (0.52)	5 (0.48)	2 (1.25)	0 (0.00)
Sarcoma	16 (0.36)	10 (0.96)	3 (1.88)	1 (5.26)
Leukemia	18 (0.41)	8 (0.76)	3 (1.88)	0 (0.00)
Laryngeal cancer	21 (0.48)	5 (0.48)	2 (1.25)	0 (0.00)
Stomach cancer	22 (0.50)	4 (0.38)	2 (1.25)	0 (0.00)
Small intestinal cancer	11 (0.25)	9 (0.86)	1 (0.63)	0 (0.00)
Uterus carcinoma	15 (0.34)	2 (0.19)	0 (0.00)	0 (0.00)
Ovarian cancer	10 (0.23)	4 (0.38)	0 (0.00)	0 (0.00)
Kaposi sarcoma (Cutaneous)	8 (0.18)	2 (0.19)	1 (0.63)	0 (0.00)
Kaposi sarcoma (Visceral)	6 (0.14)	2 (0.19)	0 (0.00)	0 (0.00)
Brain tumor	4 (0.09)	0 (0.00)	0 (0.00)	0 (0.00)

### OS of Recipients With *De Novo* PTM After DLT

**FIGURE 5:**
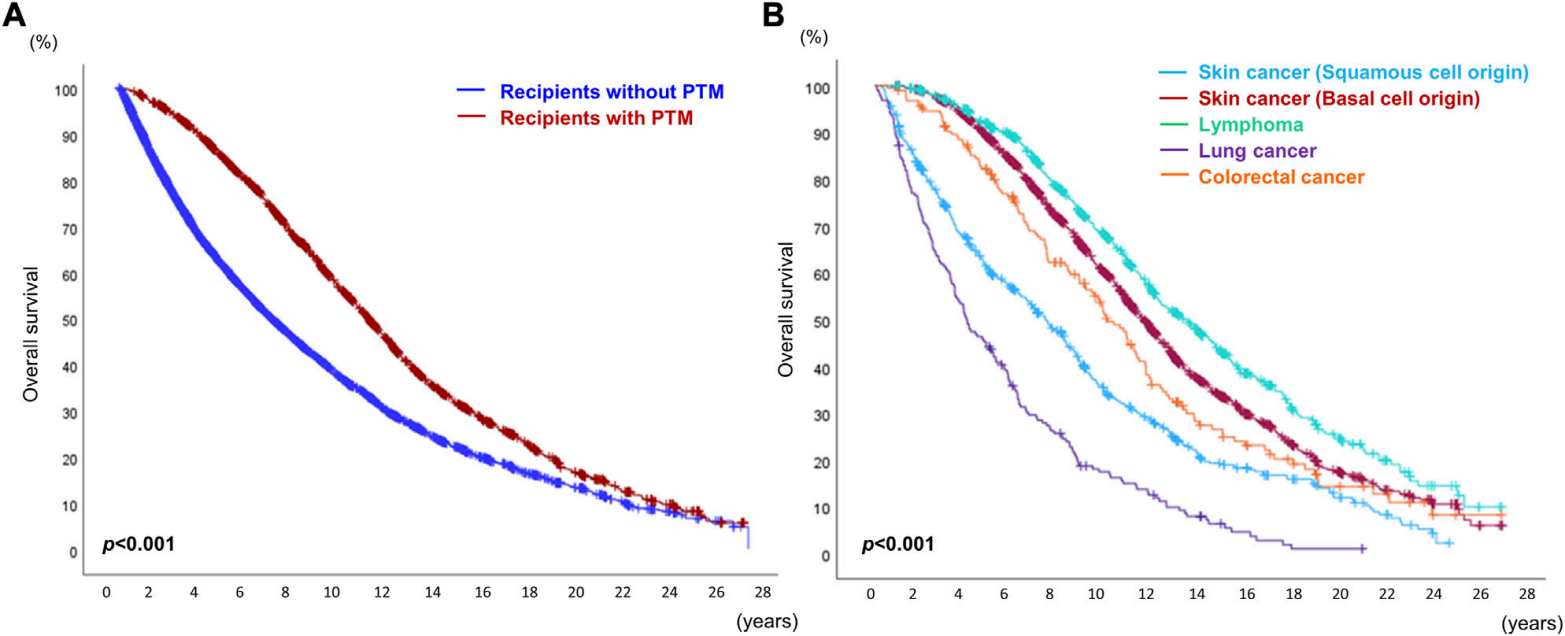
Overall survival of recipients who received double lung transplantation. **(A)** Overall survival of recipients with or without post-transplant malignancy (PTM). **(B)** Overall survival of recipients with the top five causes of *de novo* PTM after double lung transplantation.

According to the OPTN/UNOS data, the OS of all recipients who received DLT for the non-cancerous disease was 51.04% (12,216/23,935) [OS of the recipients without *de novo* PTM: 53.50% (9,794/18,306); OS of the recipients with *de novo* PTM: 43.01% (2,421/5,629). However, OS in recipients with PTM was significantly higher than that in recipients without PTM (Figure 5A). And the median and mean survival periods were significantly longer in the recipients with PTM group [median, recipients without PTM: 36.67 months (range, 0.03–330.73) vs. recipients with PTM: 97.20 months (range, 0.90–328.50); mean, recipients without PTM: 66.11 months (SD, ±66.94) vs. recipients with PTM: 106.32 months (SD, ±73.77)].

While the 5-year and 10-year OS rates in recipients with PTM were higher than in those without PTM (5-year, without PTM 67.32% vs. with PTM 83.57%; 10-year, without PTM 57.90% vs. with PTM 62.00%), the 15-year and 20-year OS rates in recipients with PTM were lower than in those without PTM (15-year, without PTM 54.68% vs. with PTM 48.80%; 20-year, without PTM 53.77% vs. with PTM 44.45%).

Among the top five causes of PTM (SCC, BCC lymphoma, lung cancer, and colorectal cancer), the OS rate of recipients with lymphoma was the highest, and that of those with lung cancer was the lowest (*p* < 0.001). The OS of recipients with SCC was worse than that of those with BCC (Figure 5B). Kaposi sarcoma (visceral type) showed the worst prognosis among the 27 different types of PTM (Supplementary Figure S2).

Age at transplantation, smoking history, occurrence of PTM and GF were associated with OS, in univariate analysis. However, in Cox regression analysis, while the occurrence of PTM was associated with lower risk of overall mortality (HR = 0.604, 95% CI: 0.575–0.635, *p* < 0.001) after adjustment for age (continuous), sex, and smoking history (non-smoker vs. smoker), the occurrence of GF was associated with higher risk of overall mortality (HR = 3.093, 95% CI: 2.936–3.257, *p* < 0.001) (Table 4).

**TABLE 4:** Factors associated with overall survival in recipients who received double lung transplantation for non-cancerous disease.

Variables	Total (*n* = 23,935)	Univariate analysis			Multivariate analysis		
		HR	95%-CI	*p*-value	HR	95%-CI	*p*-value
Age at transplantation (mean, ±SD)	51.91 ± 4.95	1.012	1.010–1.013	<0.001	1.017	1.015–1.019	<0.001
Gender (*n*, %)				0.069			0.006
Male	13,768 (57.52)	1*			1*		
Female	10,167 (42.48)	0.967	0.932–1.003		0.943	0.905–0.983	
Smoking history (*n*, %)				<0.001			0.095
Non-smoker	9,148 (38.22)	1*			1*		
Smoker	11,129 (46.50)	1.151	0.938–0.986		0.961	0.916–1.007	
Unknown	3,658 (15.28)	0.888			0.547	0.498–0.600	
Occurrence of post-transplant malignancy (*n*, %)				<0.001			<0.001
No	18,306 (76.48)	1*			1*		
Yes	5,629 (23.52)	0.586	0.562–0.610		0.604	0.575–0.635	
Occurrence of graft failure (*n*, %)				<0.001			<0.001
No	21,619 (90.32)	1*			1*		
Yes	2,316 (9.68)	2.541	2.420–2.667		3.093	2.936–3.257	

*reference

### Landmark Analysis for OS in Recipients With or Without PTM

**FIGURE 6:**
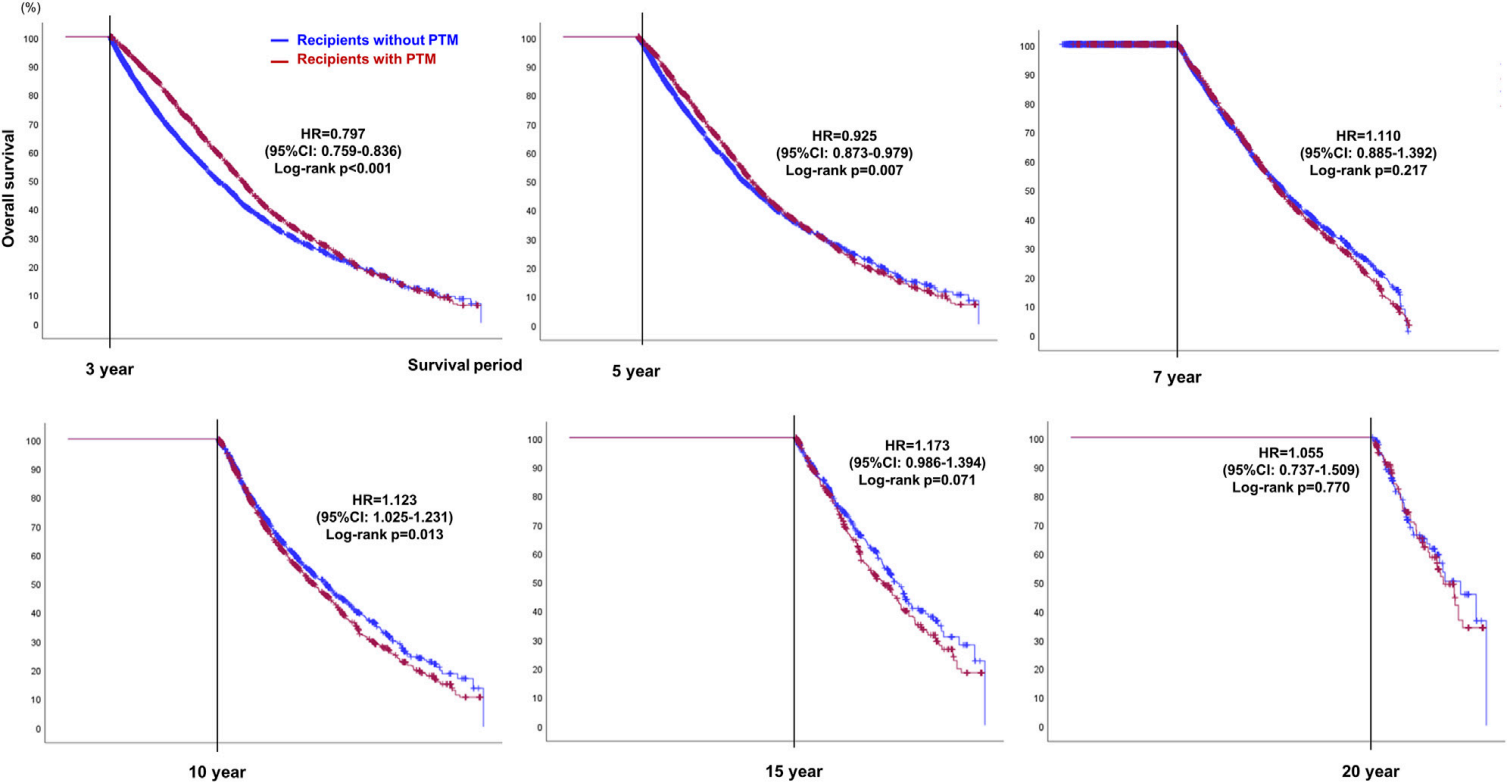
Landmark analysis plots showing OS at 3, 5, 7, 10, 15, and 20-year landmark time points in recipients with or without post-transplant malignancy (PTM) after double lung transplantation.

To compensate for the immortal time bias of PTM, OS was calculated using landmark analysis (Table 5; Figure 6). Using 3 and 5 years as the landmark time points, the OS in recipients with PTM was found to be significantly better than those without PTM (3 years, HR = 0.797, 95% CI: 0.759–0.836, *p* < 0.001; 5 years, HR = 0.925, 95% CI: 0.873–0.979, *p* = 0.007). However, at the 7-year landmark time point, the difference in OS between the two groups disappeared (*p* = 0.217), and after 10 years of surveillance, the OS in recipients without PTM was better (HR = 1.123, 95% CI: 1.025–1.231, *p* = 0.013). However, after 15 years, there was no statistical difference in OS between the two groups (15 years, HR = 1.173, 95% CI: 0.986–1.394, *p* = 0.071; 20 years, HR = 1.055, 95% CI: 0.737–1.509, *p* = 0.770).

**TABLE 5:** Number of recipients and the occurrence of post-transplant malignancy (PTM).

Overall survival (*n*, %)	Total (*n* = 23,935)	Recipients without PTM (*n* = 18,306)	Recipients with PTM (*n* = 5,629)
No landmark			
Number of death events	11,719 (48.96)	8,512 (46.50)	3,208 (56.99)
Median (month, range)	48.67 (0.03–330.73)	36.67 (0.03–330.73)	97.20 (0.90–328.50)
Mean (month, ±SD)	66.11 ± 66.94	53.74 ± 27.62	106.32 ± 73.77
5-year	75.01%	67.32%	83.57%
10-year	58.86%	57.90%	62.00%
15-year	53.29%	54.68%	48.80%
20-year	51.58%	53.77%	44.45%
3-year landmark	18,782 (78.47)	13,754 (75.13)	5,028 (89.32)
Number of death events	6,758 (28.23)	4,050 (22.12)	2,708 (48.10)
5-year landmark	16,742 (69.95)	12,201 (66.65)	4,541 (80.67)
Number of death events	4,718 (19.71)	2,497 (13.64)	2,221 (39.46)
7-year landmark	15,323 (64.02)	12,289 (67.13)	4,034 (71.66)
Number of death events	3,299 (13.78)	1,585 (8.66)	1,714 (30.45)
10-year landmark	13,436 (56.14)	10,303 (56.28)	3,133 (55.66)
Number of death events	1,412 (5.90)	599 (3.27)	813 (14.44)
15-year landmark	12,349 (51.59)	9,834 (53.72)	2,515 (44.68)
Number of death events	325 (1.36)	130 (0.71)	195 (3.46)

### Comparison of Survival Outcomes Depending on the Number of PTMs

**FIGURE 7:**
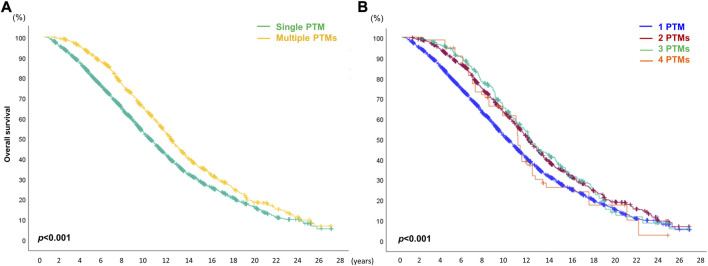
Overall survival (OS) depends on the number of *de novo* post-transplant malignancies (PTMs) after double lung transplantation. **(A)** Comparison of OS between single and multiple PTM. **(B)** Comparison of OS depending on the number of PTMs.

When the patients were divided into two cohorts, single and multiple PTM groups, the survival outcome in recipients with multiple PTMs was significantly better than that of recipients with single PTMs (*p* < 0.001). However, there was no statistically significant difference in the number of PTMs among the recipients in the multiple PTM group (*p* = 0.375) (Figure 7).

### Causes of Death in Recipients Who had Received DLT

Among the 23,935 recipients who received DLT, 11,719 recipients (48.96%) died from 74 different causes of death. The main categories of causes of death in recipients who received DLT were as follows: infection (with 13 subcategories), cardiovascular cause (with 11 subcategories), graft failure (with 8 subcategories), pulmonary cause (with 7 subcategories), malignancy (with 6 subcategories), hemorrhagic (with 6 subcategories), cerebrovascular cause (with 5 subcategories).

**FIGURE 8:**
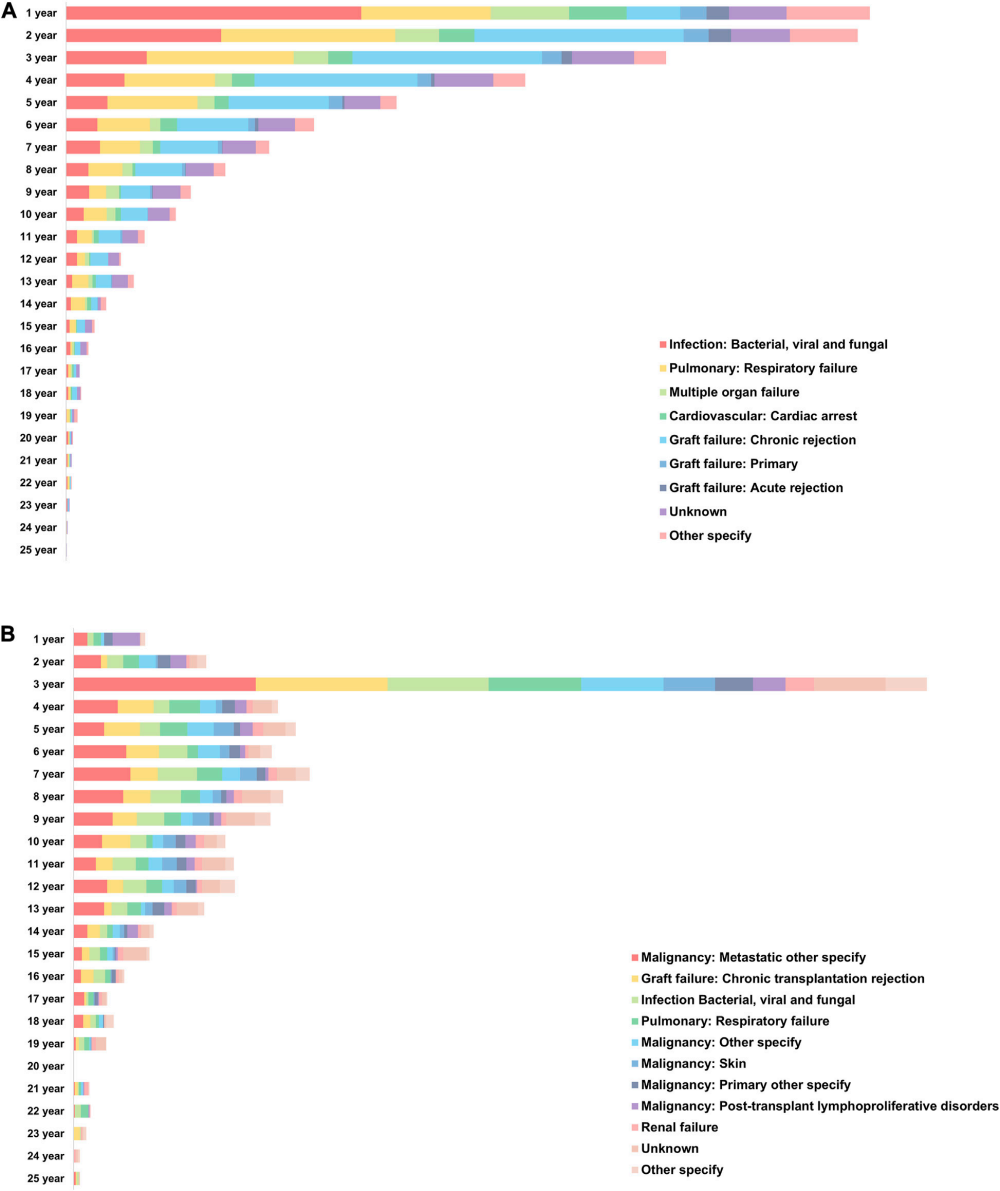
Causes of death in recipients who had received double lung transplantation. **(A)** Annual causes of death in recipients without *de novo* malignancy after double lung transplantation. **(B)** Annual causes of death in recipients with *de novo* malignancy after double lung transplantation.

The mortality rate in recipients without PTM was highest within 1 year after DLT, whereas that in recipients with PTM was highest after 3 years of DLT (Figure 8). Although graft failure was the most common cause of death in recipients without PTM, infection (including bacterial, viral, and fungal) was the most common cause of death during the first year after DLT. On the other hand, the most common cause of death in the recipients with PTM was a metastatic malignancy, which occurred most frequently in the 3 years after DLT (Supplementary Figures S3, S4).

## Discussion

Our study’s major findings include: 1) around one-fourth of the recipients who underwent DLT for non-cancerous diseases experienced PTM, with 27 different PTMs occurring during the follow-up period; 2) annual and cumulative risks of each PTM varied based on elapsed time post-DLT, with the highest PTM incidence in the second year after transplantation; 3) PTM incidence differed among age groups, particularly post-transplant lung cancer, which had the highest incidence in the 70–79 age group; 4) one in five recipients with PTM after DLT was diagnosed with multiple PTMs (up to four different types), with the most common tumor types differing based on the order of occurrence; 5) OS after DLT was better in recipients with PTM than those without PTM at the 3-year, and 5-year landmark time points and in recipients diagnosed with multiple PTMs rather than a single PTM.

Organ transplantation has increased, and survival outcomes have improved due to advancements in immunosuppressive therapy [22, 23]. However, *de novo* malignancy development post-transplantation, mainly related to immunosuppressive therapy [17, 24]. In the context of lung transplantation, although the immunosuppressive protocols are similar for both single and bilateral transplantations, our study exclusively focused on DLT. This approach was adopted to mitigate potential confounding factors such as the presence of latent lung cancer in the native lung or underlying conditions like pulmonary fibrosis that could elevate the risk of lung cancer [25, 26].

Major PTM incidences after DLT was highest in the second-year post-transplantation. However, lymphoma was most frequent at the first year than second year. Lymphoma, a post-transplant lymphoproliferative disease, typically occurs within 4–6 months after hematopoietic stem cell transplantation and mainly after the first year of solid organ transplantation [27–29]. Notably, lymphoma post-solid organ transplantation occurs 11.8-fold more frequently than in the non-transplant population (*p* < 0.001), and the age-stratified relative risk is higher in children under 10 years old and adults over 60 years. This PTM is often life-threatening, with a higher risk in heart, lung, intestinal, and multi-organ transplants [30–32]. The occurrence of post-transplant lymphoma is strongly associated with immunosuppressants, such as FK506, OKT3, and ATG [33, 34].

After DLT for non-cancerous diseases, approximately 24% of all recipients were diagnosed with PTMs in their lifetimes, with one-fifth of them being diagnosed multiple times. There were four types of post-transplant skin cancer, including SCC, BCC, melanoma, and cutaneous Kaposi sarcoma. While BCC is more prevalent than SCC in the general population at a 4:1 ratio, where SCC occurs more frequently in transplant patients with an incidence rate 65- to 250-fold higher [35]. Particularly, SCC in organ transplant recipients shows a worse prognosis with nine times higher cancer-specific mortality than in the general population [36–38]. In our study, post-transplant SCC was 3-fold higher than BCC after DLT, and the OS of recipients with BCC was better than that of those with SCC which was similar to the trend observed in the general population [39].

SCC was the most common type of PTM in most age groups, and lymphoma was the most prevalent only in the 19–29 age group. Colorectal cancer ranked as the 5th most common PTM after DLT and mainly occurred within 1 year after DLT in recipients in their 50s. After DLT, the risk of developing lymphoma and lung cancer was highest within the first year, while bladder cancer was most likely to occur 8 years after DLT. Other types of PTM occurred mainly in the second year after transplantation, with the incidence gradually decreasing over time. Interestingly, lung cancer was the 4th most common PTM after DLT, despite recipients having received bilateral allogenic lung transplantation. The incidence rates of lung cancer after DLT were only 2%–3% in most age groups, and its incidence was the highest at 8% in the 70–79 age group. While the incidence of lung cancer in the general population gradually increases with age, the recipients who received DLT showed lower occurrence rates until their 60s, which then rapidly increased in their 70s [40, 41].

Although immunosuppressive therapy after solid organ transplantation is necessary to prevent complications after transplantation [6, 42, 43]. However, long-term immunosuppression may promote cancer progression, whether it is a pre-existing or new lesion and the risk of PTM is increased approximately 3- to 4-fold compared with the general population [44–46]. A conventional protocol for maintenance immunosuppressive therapy for lung transplantation is the “triple regimen,” which includes a calcineurin inhibitor (cyclosporine or tacrolimus), antiproliferative agents (azathioprine, mycophenolate, sirolimus, and everolimus), and corticosteroids. Tacrolimus has a pro-oncogenic effect by producing transforming growth factor β1 [47], and azathioprine is known to increase the risk of skin cancers after organ transplantation, especially SCC [48, 49]. Cyclosporine use is also associated with lymphoma and skin cancer [50]. And the use of Voriconazole increases the risk for cutaneous SCC among solid organ transplant recipients [51, 52]. However, the association between mycophenolate mofetil and increased cancer incidence after transplantation is unclear. Moreover, sirolimus is known to have both an anticancer effect (by targeting mTOR) and an immunosuppressive effect [53]. To summarize, different PTMs occur depending on the regimen of immunosuppressive agents [21, 28, 54–56]. However, information on PTM remains insufficient, and there are no guidelines for modified immunosuppressive therapy that can minimize the occurrence of PTMs.

In this study, we found that recipients with PTM had significantly better survival outcomes than those without PTM. However, since an earlier study had reported significantly lower 1-year and 3-year survival rates for patients with PTM [57], we conducted a landmark survival analysis to shed more light on this discrepancy. We assumed this was because of the immortal time bias, which means that longer recipients have a higher chance of being diagnosed with PTM. To compensate for this error, which refers to a bias that can occur in observational studies when the time between a defined event (e.g., transplantation) and the start of follow-up (e.g., diagnosis of PTM) is not considered [58], we performed landmark analysis with 3, 5, 7, 10, 15, and 20 years as the landmark time points. Recipients with PTM had better short-term survival (3–5 years) but worse long-term survival (10 years and beyond). Immunosuppressive therapy may contribute to PTM while preventing graft rejection. Graft failure was a major cause of death in recipients without PTM. Factors like age and comorbidities may have a greater impact on long-term survival. Beyond 15–20 years, there was no statistical difference in survival, possibly due to other factors and decreased statistical power.

The major limitation of this study is that not all patients had the same length of follow-up period and actual incidence of PTM could not be calculated for individuals who did not reach the 1-year follow-up after transplantation. And although at least 10 years of follow-up results were investigated for most PTMs, only 9, 6, and 4 years of follow-up results were available for leukemia, Kaposi sarcoma, and brain tumor, respectively. Another limitation of this study is that we did not completely correct for the higher chance of developing cancer over time, even though we performed a landmark analysis. However, this study provided general information on PTMs in recipients who received DLT for non-cancerous diseases, offering a comprehensive landscape in this field.

In conclusion, the types and characteristics of PTMs in recipients who received DLT for non-cancerous diseases were highly diverse, and the incidence varied according to age and duration after transplantation. Additionally, the survival outcomes showed significant differences depending on the existence or types of PTM. Nevertheless, we were able to identify the specific times at which each type of PTM frequently occurred. By gaining a more comprehensive understanding of the characteristics of PTMs in recipients who have undergone DLT, it may become possible to predict with greater accuracy the specific types of PTM that are most likely to occur over time and to facilitate their early detection. Such insights can potentially revolutionize our approach to monitoring and managing PTMs in DLT recipients, ultimately leading to improved clinical outcomes and a better quality of life for those who have undergone this procedure.

## Data Availability

The data analyzed in this study is subject to the following licenses/restrictions: The data that support the findings of this study are available from the corresponding author upon reasonable request. Requests to access these datasets should be directed to ychae@nm.org.

## Footnotes

1
www.unos.org

## Abbreviations

DLT, double lung transplantation; OPTN, Organ Procurement and Transplantation Network; PTM, post-transplant malignancy; UNOS, United Network for Organ Sharing.

## References

[1] Wolfe RA Roys EC Merion RM. Trends in Organ Donation and Transplantation in the United States, 1999-2008. Am J Transplant (2010) 10(2):961–72. 10.1111/j.1600-6143.2010.03021.x 20420646

[2] Israni AK. OPTN/SRTR 2020 Annual Data Report: Introduction. Am J Transplant (2022) 22(2):11–20. 10.1111/ajt.16974 35266612

[3] Thabut G Christie JD Ravaud P Castier Y Brugière O Fournier M Survival After Bilateral Versus Single Lung Transplantation for Patients With Chronic Obstructive Pulmonary Disease: A Retrospective Analysis of Registry Data. Lancet (2008) 371(9614):744–51. 10.1016/S0140-6736(08)60344-X 18313503

[4] Valapour M Lehr CJ Skeans MA Smith JM Miller E Goff R OPTN/SRTR 2020 Annual Data Report: Lung. Am J Transplant (2022) 22(2):438–518. 10.1111/ajt.16991 35266615

[5] Chinen J Buckley RH. Transplantation Immunology: Solid Organ and Bone Marrow. J Allergy Clin Immunol (2010) 125(2):S324–35. 10.1016/j.jaci.2009.11.014 20176267 PMC2857984

[6] Duncan MD Wilkes DS. Transplant-Related Immunosuppression: A Review of Immunosuppression and Pulmonary Infections. Proc Am Thorac Soc (2005) 2(5):449–55. 10.1513/pats.200507-073JS 16322599 PMC2713333

[7] Slepicka PF Yazdanifar M Bertaina A. Harnessing Mechanisms of Immune Tolerance to Improve Outcomes in Solid Organ Transplantation: A Review. Front Immunol (2021) 12:688460. 10.3389/fimmu.2021.688460 34177941 PMC8222735

[8] Snell GI Westall GP. Immunosuppression for Lung Transplantation: Evidence to Date. Drugs (2007) 67(11):1531–9. 10.2165/00003495-200767110-00002 17661526

[9] Scheffert JL Raza K. Immunosuppression in Lung Transplantation. J Thorac Dis (2014) 6(8):1039–53. 10.3978/j.issn.2072-1439.2014.04.23 25132971 PMC4133546

[10] Taylor AL Watson CJE Bradley JA. Immunosuppressive Agents in Solid Organ Transplantation: Mechanisms of Action and Therapeutic Efficacy. Crit Rev Oncol Hematol (2005) 56(1):23–46. 10.1016/j.critrevonc.2005.03.012 16039869

[11] Snell GI Westall GP Paraskeva MA. Immunosuppression and Allograft Rejection Following Lung Transplantation: Evidence to Date. Drugs (2013) 73(16):1793–813. 10.1007/s40265-013-0136-x 24142409

[12] Wiseman AC. Immunosuppressive Medications. Clin J Am Soc Nephrol (2016) 11(2):332–43. 10.2215/CJN.08570814 26170177 PMC4741049

[13] Zeyda M Geyeregger R Poglitsch M Weichhart T Zlabinger GJ Koyasu S Impairment of T Cell Interactions With Antigen-Presenting Cells by Immunosuppressive Drugs Reveals Involvement of Calcineurin and NF-KappaB in Immunological Synapse Formation. J Leukoc Biol (2007) 81(1):319–27. 10.1189/jlb.0606378 17038582

[14] Tatapudi VS Montgomery RA. Therapeutic Modulation of the Complement System in Kidney Transplantation: Clinical Indications and Emerging Drug Leads. Front Immunol (2019) 10:2306. 10.3389/fimmu.2019.02306 31632397 PMC6779821

[15] Dunn GP Bruce AT Ikeda H Old LJ Schreiber RD. Cancer Immunoediting: From Immunosurveillance to Tumor Escape. Nat Immunol (2002) 3(11):991–8. 10.1038/ni1102-991 12407406

[16] Tie Y Tang F Wei YQ Wei XW. Immunosuppressive Cells in Cancer: Mechanisms and Potential Therapeutic Targets. J Hematol Oncol (2022) 15(1):61. 10.1186/s13045-022-01282-8 35585567 PMC9118588

[17] Engels EA Pfeiffer RM Fraumeni JF Kasiske BL Israni AK Snyder JJ Spectrum of Cancer Risk Among US Solid Organ Transplant Recipients. JAMA (2011) 306(17):1891–901. 10.1001/jama.2011.1592 22045767 PMC3310893

[18] Wimmer CD Rentsch M Crispin A Illner WD Arbogast H Graeb C The Janus Face of Immunosuppression - De Novo Malignancy After Renal Transplantation: The Experience of the Transplantation Center Munich. Kidney Int (2007) 71(12):1271–8. 10.1038/sj.ki.5002154 17332737

[19] Buell JF Gross TG Woodle ES. Malignancy After Transplantation. Transplantation (2005) 80(2):S254–64. 10.1097/01.tp.0000186382.81130.ba 16251858

[20] Friman TK Jäämaa-Holmberg S Åberg F Helanterä I Halme M Pentikäinen MO Cancer Risk and Mortality After Solid Organ Transplantation: A Population-Based 30-Year Cohort Study in Finland. Int J Cancer (2022) 150(11):1779–91. 10.1002/ijc.33934 35041762 PMC9306582

[21] Dantal J Soulillou JP. Immunosuppressive Drugs and the Risk of Cancer After Organ Transplantation. N Engl J Med (2005) 352(13):1371–3. 10.1056/NEJMe058018 15800234

[22] Yusen RD Christie JD Edwards LB Kucheryavaya AY Benden C Dipchand AI The Registry of the International Society for Heart and Lung Transplantation: Thirtieth Adult Lung and Heart-Lung Transplant Report--2013; Focus Theme: Age. J Heart Lung Transplant (2013) 32(10):965–78. 10.1016/j.healun.2013.08.007 24054805

[23] Rana A Godfrey EL. Outcomes in Solid-Organ Transplantation: Success and Stagnation. Tex Heart Inst J (2019) 46(1):75–6. 10.14503/THIJ-18-6749 30833851 PMC6379008

[24] Chapman JR Webster AC Wong G. Cancer in the Transplant Recipient. Cold Spring Harb Perspect Med (2013) 3(7):a015677. 10.1101/cshperspect.a015677 23818517 PMC3685882

[25] Meyer EC Liebow AA. Relationship Of Interstitial Pneumonia Honeycombing and Atypical Epithelial Proliferation To Cancer Of The Lung. Cancer (1965) 18:322–51. 10.1002/1097-0142(196503)18:3<322::aid-cncr2820180310>3.0.co;2-j 14264034

[26] Ballester B Milara J Cortijo J. Idiopathic Pulmonary Fibrosis and Lung Cancer: Mechanisms and Molecular Targets. Int J Mol Sci (2019) 20(3):593. 10.3390/ijms20030593 30704051 PMC6387034

[27] Novoa-Takara L Perkins SL Qi D Shidham VB Vesole DH Hariharan S Histogenetic Phenotypes of B Cells in Posttransplant Lymphoproliferative Disorders by Immunohistochemical Analysis Correlate With Transplant Type: Solid Organ vs Hematopoietic Stem Cell Transplantation. Am J Clin Pathol (2005) 123(1):104–12. 10.1309/dw2tw2087bxl2brk 15762285

[28] Opelz G Döhler B. Lymphomas After Solid Organ Transplantation: A Collaborative Transplant Study Report. Am J Transplant (2004) 4(2):222–30. 10.1046/j.1600-6143.2003.00325.x 14974943

[29] Curtis RE Travis LB Rowlings PA Socié G Kingma DW Banks PM Risk of Lymphoproliferative Disorders After Bone Marrow Transplantation: A Multi-Institutional Study. Blood (1999) 94(7):2208–16. 10.1182/blood.V94.7.2208.419k21_2208_2216 10498590

[30] Clarke CA Morton LM Lynch C Pfeiffer RM Hall EC Gibson TM Risk of Lymphoma Subtypes After Solid Organ Transplantation in the United States. Br J Cancer (2013) 109(1):280–8. 10.1038/bjc.2013.294 23756857 PMC3708563

[31] Gottschalk S Rooney CM Heslop HE. Post-Transplant Lymphoproliferative Disorders. Annu Rev Med (2005) 56:29–44. 10.1146/annurev.med.56.082103.104727 15660500

[32] Haldas J Wang W Lazarchick J. Post-Transplant Lymphoproliferative Disorders: T-Cell Lymphoma Following Cardiac Transplant. Leuk Lymphoma (2002) 43(2):447–50. 10.1080/10428190290006332 11999587

[33] Cockfield SM. Identifying the Patient at Risk for Post-Transplant Lymphoproliferative Disorder. Transpl Infect Dis (2001) 3(2):70–8. 10.1034/j.1399-3062.2001.003002070.x 11395972

[34] Ogata T Yamasaki Y. Ultra-High-Resolution Scanning Electron Microscopic Studies on the Sarcoplasmic Reticulum and Mitochondria of the Rat Intrafusal Muscle Fibers. Part II: The Extracapsular Region. Arch Histol Cytol (1992) 55(2):117–24. 10.1679/aohc.55.117 1497943

[35] Bouwes Bavinck JN Euvrard S Naldi L Nindl I Proby CM Neale R Keratotic Skin Lesions and Other Risk Factors Are Associated With Skin Cancer in Organ-Transplant Recipients: A Case-Control Study in the Netherlands, United Kingdom, Germany, France, and Italy. J Invest Dermatol (2007) 127(7):1647–56. 10.1038/sj.jid.5700776 17380113 PMC2478722

[36] Bibee K Swartz A Sridharan S Kurten CHL Wessel CB Skinner H Cutaneous Squamous Cell Carcinoma in the Organ Transplant Recipient. Oral Oncol (2020) 103:104562. 10.1016/j.oraloncology.2019.104562 32065978 PMC7217490

[37] Manyam BV Gastman B Zhang AY Reddy CA Burkey BB Scharpf J Inferior Outcomes in Immunosuppressed Patients With High-Risk Cutaneous Squamous Cell Carcinoma of the Head and Neck Treated With Surgery and Radiation Therapy. J Am Acad Dermatol (2015) 73(2):221–7. 10.1016/j.jaad.2015.04.037 26028524

[38] Howard MD Su JC Chong AH. Skin Cancer Following Solid Organ Transplantation: A Review of Risk Factors and Models of Care. Am J Clin Dermatol (2018) 19(4):585–97. 10.1007/s40257-018-0355-8 29691768

[39] Rees JR Zens MS Celaya MO Riddle BL Karagas MR Peacock JL. Survival After Squamous Cell and Basal Cell Carcinoma of the Skin: A Retrospective Cohort Analysis. Int J Cancer (2015) 137(4):878–84. 10.1002/ijc.29436 25598534 PMC4478145

[40] de Groot PM Wu CC Carter BW Munden RF. The Epidemiology of Lung Cancer. Transl Lung Cancer Res (2018) 7(3):220–33. 10.21037/tlcr.2018.05.06 30050761 PMC6037963

[41] Akgün KM Crothers K Pisani M. Epidemiology and Management of Common Pulmonary Diseases in Older Persons. J Gerontol A Biol Sci Med Sci (2012) 67(3):276–91. 10.1093/gerona/glr251 22337938 PMC3297767

[42] Pilch NA Bowman LJ Taber DJ. Immunosuppression Trends in Solid Organ Transplantation: The Future of Individualization, Monitoring, and Management. Pharmacotherapy (2021) 41(1):119–31. 10.1002/phar.2481 33131123 PMC8778961

[43] Mahmud N Klipa D Ahsan N. Antibody Immunosuppressive Therapy in Solid-Organ Transplant: Part I. MAbs (2010) 2(2):148–56. 10.4161/mabs.2.2.11159 20150766 PMC2840233

[44] Webster AC Craig JC Simpson JM Jones MP Chapman JR. Identifying High Risk Groups and Quantifying Absolute Risk of Cancer After Kidney Transplantation: A Cohort Study of 15,183 Recipients. Am J Transpl (2007) 7(9):2140–51. 10.1111/j.1600-6143.2007.01908.x 17640312

[45] Lindelöf B Sigurgeirsson B Gäbel H Stern RS. Incidence of Skin Cancer in 5356 Patients Following Organ Transplantation. Br J Dermatol (2000) 143(3):513–9. 10.1046/j.1365-2133.2000.03703.x 10971322

[46] Adami J Gäbel H Lindelöf B Ekström K Rydh B Glimelius B Cancer Risk Following Organ Transplantation: A Nationwide Cohort Study in Sweden. Br J Cancer (2003) 89(7):1221–7. 10.1038/sj.bjc.6601219 14520450 PMC2394311

[47] Maluccio M Sharma V Lagman M Vyas S Yang H Li B Tacrolimus Enhances Transforming Growth Factor-Beta1 Expression and Promotes Tumor Progression. Transplantation (2003) 76(3):597–602. 10.1097/01.TP.0000081399.75231.3B 12923450

[48] Vos M Plasmeijer EI van Bemmel BC van der Bij W Klaver NS Erasmus ME Azathioprine to Mycophenolate Mofetil Transition and Risk of Squamous Cell Carcinoma After Lung Transplantation. J Heart Lung Transpl (2018) 37(7):853–9. 10.1016/j.healun.2018.03.012 29680587

[49] Jiyad Z Olsen CM Burke MT Isbel NM Green AC. Azathioprine and Risk of Skin Cancer in Organ Transplant Recipients: Systematic Review and Meta-Analysis. Am J Transpl (2016) 16(12):3490–503. 10.1111/ajt.13863 27163483

[50] Parekh K Trulock E Patterson GA. Use of Cyclosporine in Lung Transplantation. Transpl Proc (2004) 36(2):318S–322S. 10.1016/j.transproceed.2004.01.056 15041361

[51] Kuklinski LF Li S Karagas MR Weng WK Kwong BY. Effect of Voriconazole on Risk of Nonmelanoma Skin Cancer After Hematopoietic Cell Transplantation. J Am Acad Dermatol (2017) 77(4):706–12. 10.1016/j.jaad.2017.06.032 28780363 PMC5877457

[52] Williams K Mansh M Chin-Hong P Singer J Arron ST. Voriconazole-Associated Cutaneous Malignancy: A Literature Review on Photocarcinogenesis in Organ Transplant Recipients. Clin Infect Dis (2014) 58(7):997–1002. 10.1093/cid/cit940 24363331 PMC6276938

[53] Vignot S Faivre S Aguirre D Raymond E. mTOR-Targeted Therapy of Cancer With Rapamycin Derivatives. Ann Oncol (2005) 16(4):525–37. 10.1093/annonc/mdi113 15728109

[54] Birkeland SA Storm HH Lamm LU Barlow L Blohmé I Forsberg B Cancer Risk After Renal Transplantation in the Nordic Countries, 1964-1986. Int J Cancer (1995) 60(2):183–9. 10.1002/ijc.2910600209 7829213

[55] Swinnen LJ Costanzo-Nordin MR Fisher SG O'Sullivan EJ Johnson MR Heroux AL Increased Incidence of Lymphoproliferative Disorder After Immunosuppression With the Monoclonal Antibody OKT3 in Cardiac-Transplant Recipients. N Engl J Med (1990) 323(25):1723–8. 10.1056/NEJM199012203232502 2100991

[56] Asleh R Alnsasra H Habermann TM Briasoulis A Kushwaha SS. Post-Transplant Lymphoproliferative Disorder Following Cardiac Transplantation. Front Cardiovasc Med (2022) 9:787975. 10.3389/fcvm.2022.787975 35282339 PMC8904724

[57] Magruder JT Crawford TC Grimm JC Kim B Shah AS Bush EL Risk Factors for De Novo Malignancy Following Lung Transplantation. Am J Transplant (2017) 17(1):227–38. 10.1111/ajt.13925 27321167

[58] Yadav K Lewis RJ. Immortal Time Bias in Observational Studies. JAMA (2021) 325(7):686–7. 10.1001/jama.2020.9151 33591334

