# Supplementary figures and images for: A Comprehensive Landscape of De Novo Malignancy After Double Lung Transplantation
Source: Transpl Int. 2023 Aug 17;36:11552. doi: 10.3389/ti.2023.11552 (PMC10468575; doi:10.3389/ti.2023.11552)

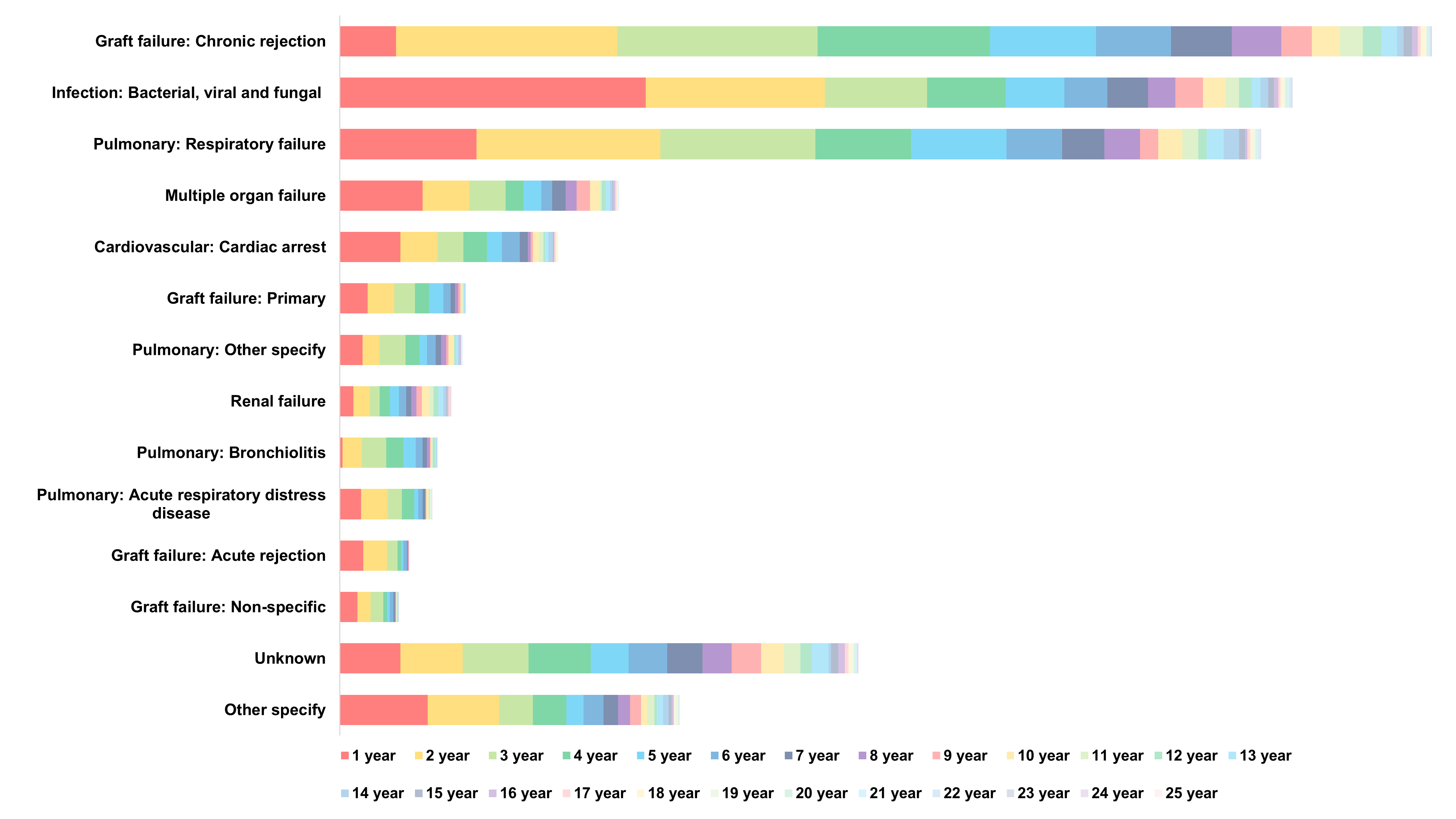

Supplement: Supplementary file 1 [file Image3.tif]

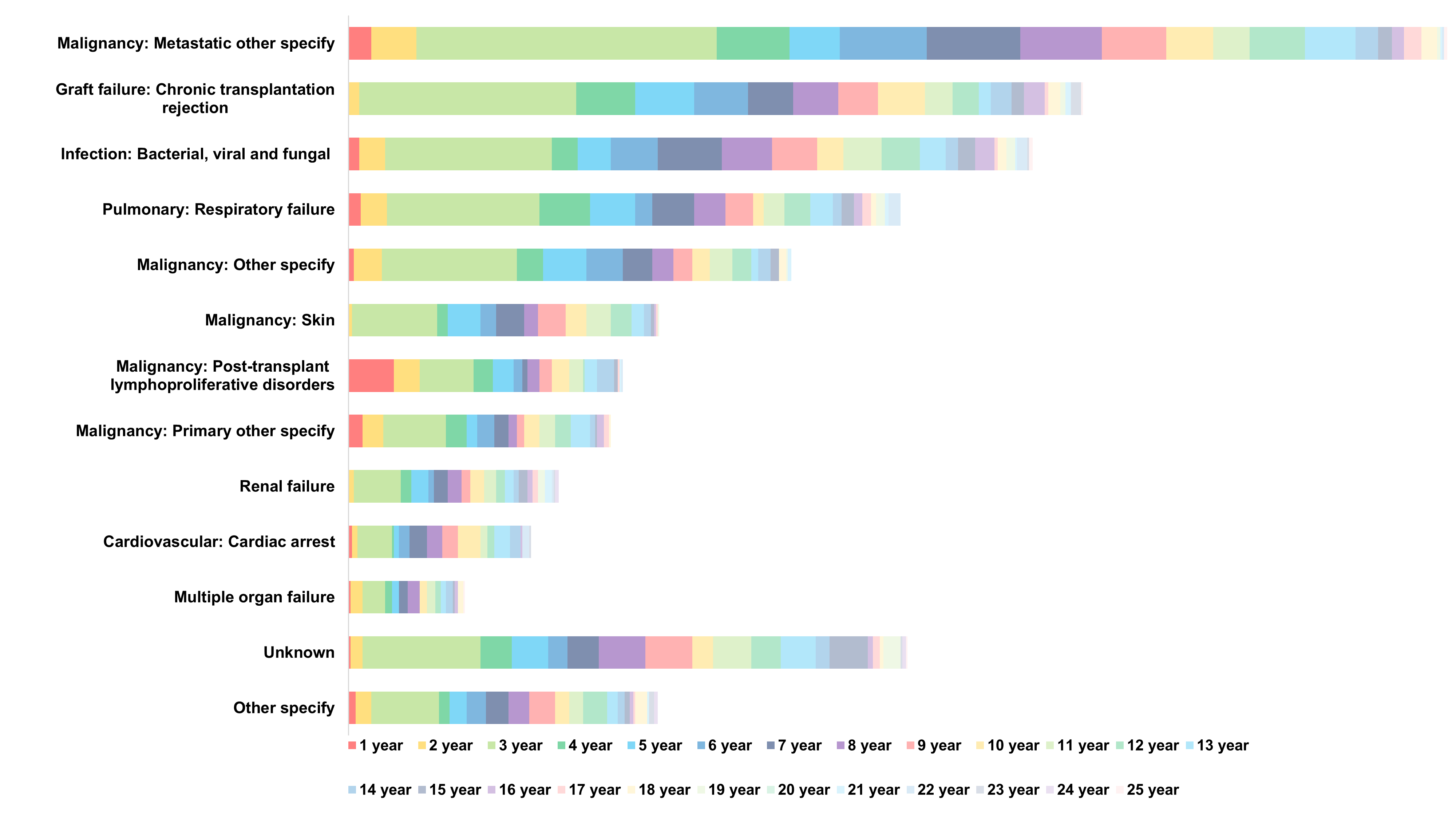

Supplement: Supplementary file 2 [file Image4.tif]

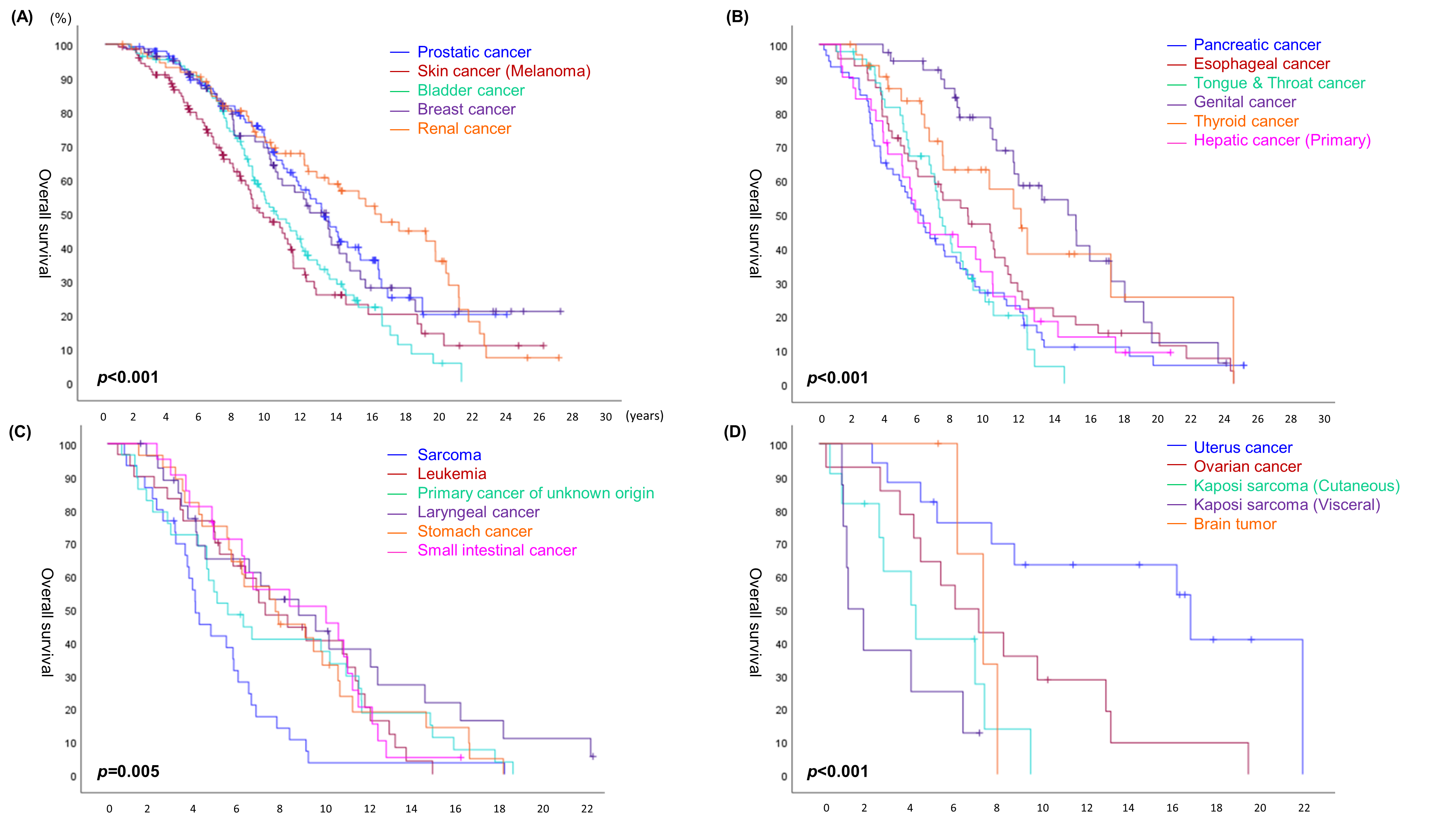

Supplement: Supplementary file 3 [file Image2.TIF]

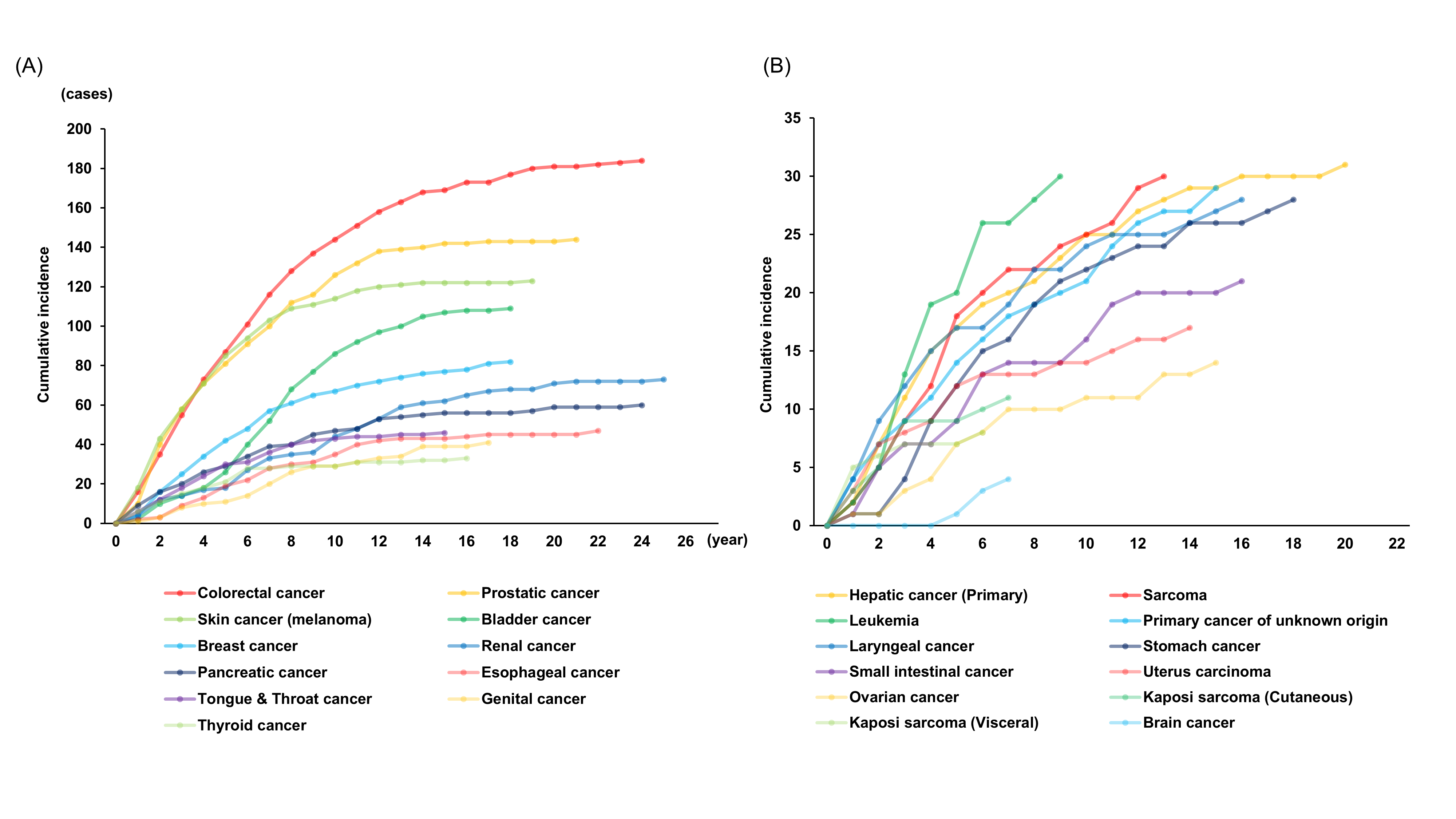

Supplement: Supplementary file 4 [file Image1.TIF]
