# Supplementary material for: A Comprehensive Landscape of De Novo Malignancy After Double Lung Transplantation
Source: Transpl Int. 2023 Aug 17;36:11552. doi: 10.3389/ti.2023.11552 (PMC10468575; doi:10.3389/ti.2023.11552)
Supplement: Supplementary file 5 [file DataSheet1.docx]

Figure S1. Cumulative risks of 5th–15th (A), 16th–27th (B) common causes of de novo malignancy after double lung transplantation.

Figure S2. Overall survival in recipients with other de novo malignancies (except for the top five) after double lung plantation; 6th–10th (A), 11th–16th (B), 17th–22nd (C), and 23rd–27th (D) common malignancies.

Figure S3. Causes of death in recipients who received double lung transplantation (DLT) without de novo post-transplant malignancy after DLT.

Figure S4. Causes of death in recipients who received double lung transplantation (DLT) with post-transplant malignancy after DLT.

Supplementary table1. Indications for double lung transplantation

|  | Total  (n=23,935) | Recipients without PTM  (n=18,306) | Recipients with PTM  (n=5,629) |
| --- | --- | --- | --- |
| Idiopathic pulmonary fibrosis/usual interstitial pneumonitis | 6,400 (26.74) | 4,687 (25.60) | 1,713 (32.14) |
| Chronic obstructive pulmonary disease (COPD)/emphysema | 5,276 (22.04) | 3,777 (20.63) | 1,499 (28.13) |
| Cystic fibrosis | 4,075 (17.03) | 3,308 (18.07) | 767 (14.39) |
| Pulmonary fibrosis (other specified cause) | 6400 (26.74) | 935 (5.11) | 263 (4.94) |
| Primary pulmonary hypertension | 5276 (22.04) | 689 (3.76) | 136 (2.55) |
| Alpha-1-antitrypsin deficiency | 4075 (17.03) | 656 (3.58) | 281 (5.27) |
| Sarcoidosis | 1198 (5.01) | 652 (356) | 156 (2.93) |
| Bronchiectasis | 825 (3.45) | 494 (2.70) | 129 (2.42) |
| Hypersensitivity pneumonitis | 937 (3.91) | 411 (2.25) | 97 (1.82) |
| Idiopathic interstitial pneumonia (IIP) (non-specified) | 808 (3.38) | 349 (1.91) | 28 (0.53) |
| Scleroderma-pulmonary hypertension | 623 (2.60) | 185 (1.01) | 32 (0.60) |
| Scleroderma-restrictive | 508 (2.12) | 171 (0.93) | 30 (0.56) |
| Acute respiratory distress syndrome (ARDS) due to COVID-19 | 377 (1.58) | 151 (0.82) | 1 (0.02) |
| Obliterative bronchiolitis (non-re-transplantation) | 217 (0.91) | 128 (0.70) | 26 (0.49) |
| Lymphangioleiomyomatosis | 201 (0.84) | 121 (0.66) | 55 (1.03) |
| Mixed connective tissue disease | 152 (0.64) | 115 (0.63) | 6 (0.11) |
| Rheumatoid disease | 154 (0.64) | 110 (0.60) | 13 (0.24) |
| Secondary pulmonary hypertension | 132 (0.55) | 109 (0.60) | 23 (0.43) |
| Lung re-transplantation/graft failure: obliterative bronchiolitis-obstructive | 145 (0.61) | 105 (0.57) | 40 (0.75) |
| Other-specified | 170 (0.71) | 99 (0.54) | 71 (1.33) |
| Lung re-transplantation/graft failure: obliterative bronchiolitis | 111 (0.46) | 71 (0.39) | 40 (0.75) |
| Occupational lung disease (other specified) | 98 (0.41) | 68 (0.37) | 30 (0.56) |
| Pulmonary fibrosis due to COVID-19 | 66 (0.28) | 66 (0.36) | 0 (0.00) |
| Pulmonary veno-occlusive disease | 73 (0.30) | 62 (0.34) | 11 (0.21) |
| Polymyositis | 58 (0.24) | 51 (0.28) | 7 (0.13) |
| Eosinophilic granuloma | 63 (0.26) | 50 (0.27) | 13 (0.24) |
| Lung re-transplantation/graft failure: primary graft failure | 80 (0.33) | 50 (0.27) | 30 (0.56) |
| Bronchiolitis obliterans organizing pneumonia (BOOP) | 55 (0.23) | 48 (0.26) | 7 (0.13) |
| ARDS/pneumonia | 49 (0.20) | 45 (0.25) | 4 (0.08) |
| Sjogren’s syndrome | 42 (0.18) | 34 (0.19) | 8 (0.15) |
| Lung re-transplantation/graft failure: obliterative bronchiolitis-restrictive | 49 (0.20) | 34 (0.19) | 15 (0.28) |
| Constrictive bronchiolitis | 36 (0.15) | 31 (0.17) | 5 (0.09) |
| Idiopathic pulmonary hemosiderosis | 33 (0.14) | 25 (0.14) | 8 (0.15) |
| Silicosis | 38 (0.16) | 25 (0.14) | 13 (0.24) |
| Secondary pulmonary fibrosis (specified cause) | 26 (0.11) | 23 (0.13) | 3 (0.06) |
| Lupus | 21 (0.09) | 20 (0.11) | 1 (0.02) |
| Eisenmenger’s syndrome: atrial septal defect | 33 (0.14) | 20 (0.11) | 13 (0.24) |
| Hermansky-Pudlak syndrome | 21 (0.09) | 19 (0.10) | 2 (0.04) |
| IIP: Acute interstitial pneumonia | 21 (0.09) | 19 (0.10) | 2 (0.04) |
| CREST syndrome (pulmonary hypertension) | 18 (0.08) | 18 (0.10) | 0 (0.00) |
| IIP: Desquamative interstitial pneumonia | 19 (0.08) | 18 (0.10) | 1 (0.02) |
| Lung re-transplantation /graft failure: other specified | 25 (0.10) | 18 (0.10) | 7 (0.13) |
| Bronchopulmonary dysplasia | 19 (0.08) | 17 (0.09) | 2 (0.04) |
| Kartagener’s syndrome | 16 (0.07) | 15 (0.08) | 1 (0.02) |
| Obstructive lung disease | 18 (0.08) | 14 (0.08) | 4 (0.08) |
| Eisenmenger’s syndrome: ventricular septal defect | 15 (0.06) | 14 (0.08) | 1 (0.02) |
| Common variable immune deficiency | 12 (0.05) | 12 (0.07) | 0 (0.00) |
| Lymphocytic interstitial pneumonitis | 14 (0.06) | 12 (0.07) | 2 (0.04) |
| Alveolar proteinosis | 11 (0.05) | 11 (0.06) | 0 (0.00) |
| Pulmonary vascular disease | 17 (0.07) | 11 (0.06) | 6 (0.11) |
| Primary ciliary dyskinesia | 13 (0.05) | 10 (0.05) | 3 (0.06) |
| Graft-versus-host disease (GVHD) | 9 (0.04) | 8 (0.04) | 1 (0.02) |
| Porto-pulmonary hypertension | 7 (0.03) | 7 (0.04) | 0 (0.00) |
| Pulmonary telangiectasia-pulmonary hypertension | 7 (0.03) | 7 (0.04) | 0 (0.00) |
| Fibro-cavitary lung disease | 7 (0.03) | 7 (0.04) | 0 (0.00) |
| Thrombo-embolic pulmonary hypertension | 6 (0.03) | 6 (0.03) | 0 (0.00) |
| Granulomatosis lung disease | 9 (0.04) | 5 (0.03) | 4 (0.08) |
| Wegener’s granuloma (restrictive) | 7 (0.03) | 5 (0.03) | 2 (0.04) |
| Lung re-transplantation /graft failure: non-specified | 6 (0.03) | 5 (0.03) | 1 (0.02) |
| Lung re-transplantation/graft failure: restrictive | 7 (0.03) | 5 (0.03) | 2 (0.04) |
| Lung re-transplantation/graft failure: acute rejection | 6 (0.03) | 5 (0.03) | 1 (0.02) |
| Lung re-transplantation/graft failure: obstructive | 8 (0.03) | 5 (0.03) | 3 (0.06) |
| Congenital: other specified | 5 (0.02) | 5 (0.03) | 0 (0.00) |
| Allergic bronchopulmonary aspergillosis | 7 (0.03) | 4 (0.02) | 3 (0.06) |
| Wegener’s granuloma-bronchiectasis | 5 (0.02) | 4 (0.02) | 1 (0.02) |
| Eisenmenger’s syndrome: patent ductus arteriosus | 4 (0.02) | 4 (0.02) | 0 (0.00) |
| Pulmonary capillary hemangiomatosis | 4 (0.02) | 4 (0.02) | 0 (0.00) |
| Congenital malformation | 3 (0.01) | 3 (0.02) | 0 (0.00) |
| CREST syndrome (restrictive) | 3 (0.01) | 3 (0.02) | 0 (0.00) |
| Hypogammaglobulinemia | 3 (0.01) | 3 (0.02) | 0 (0.00) |
| Scleroderma | 3 (0.01) | 3 (0.02) | 0 (0.00) |
| IIP: Respiratory bronchiolitis | 5 (0.02) | 3 (0.02) | 2 (0.04) |
| Inhalation burns/trauma | 3 (0.01) | 3 (0.02) | 0 (0.00) |
| Tuberous sclerosis | 2 (0.01) | 2 (0.01) | 0 (0.00) |
| Pulmonary telangiectasia-restrictive | 3 (0.01) | 2 (0.01) | 1 (0.02) |
| ABCA3 transporter mutation | 2 (0.01) | 2 (0.01) | 0 (0.00) |
| Surfactant protein deficiency | 2 (0.01) | 2 (0.01) | 0 (0.00) |
| Eisenmenger’s syndrome: multiple congenital anomaly | 2 (0.01) | 2 (0.01) | 0 (0.00) |
| Eisenmenger’s syndrome: other specified | 3 (0.01) | 2 (0.01) | 1 (0.02) |
| Pulmonary thromboembolic disease | 1 (0.00) | 1 (0.01) | 0 (0.00) |
| Schwachman-Diamond syndrome | 1 (0.00) | 1 (0.01) | 0 (0.00) |
| Fibrosing mediastinitis | 1 (0.00) | 1 (0.01) | 0 (0.00) |
| Paraneoplastic pemphigus associated Castleman disease | 1 (0.00) | 1 (0.01) | 0 (0.00) |
| Pulmonary hyalinizing granuloma | 1 (0.00) | 1 (0.01) | 0 (0.00) |
| Surfactant protein B deficiency | 1 (0.00) | 1 (0.01) | 0 (0.00) |
| Restrictive myopathy: sarcoidosis | 1 (0.00) | 1 (0.01) | 0 (0.00) |
| Amyloidosis | 1 (0.00) | 0 (0.00) | 1 (0.02) |
| Dilated myopathy: ischemic | 1 (0.00) | 0 (0.00) | 1 (0.02) |

Supplementary table 2. Causes of death in recipients with or without post-transplantation (PTM) who had received double lung transplantation

| Causes of death (n, %) | Recipients without PTM  (n=8,514) | Recipients with PTM  (n=3,208) |
| --- | --- | --- |
| Infection | 1,681 (19.74) | 428 (13.34) |
| Graft failure: chronic rejection | 1,527 (17.94) | 321 (10.01) |
| Pulmonary: respiratory failure | 1,284 (15.08) | 248 (7.73) |
| Pulmonary: others | 521 (6.12) | 81 (2.52) |
| Multiple organ failure | 386 (4.53) | 58 (1.81) |
| Cardiovascular: cardiac arrest | 302 (3.55) | 93 (2.90) |
| Cardiovascular: others | 185 (2.17) | 55 (1.71) |
| Graft failure: primary failure | 176 (2.07) | 12 (0.37) |
| Renal failure | 156 (1.83) | 94 (2.93) |
| Graft failure: others | 142 (1.67) | 24 (0.75) |
| Cerebrovascular: others | 122 (1.43) | 26 (0.81) |
| Hemorrhage | 112 (1.32) | 21 (0.65) |
| Graft failure: hyperacute or acute rejection | 103 (1.21) | 11 (0.34) |
| Cerebrovascular: stroke | 78 (0.92) | 32 (1.00) |
| Malignancy: others | 50 (0.59) | 526 (16.40) |
| Liver failure | 49 (0.58) | 30 (0.94) |
| Malignancy: metastatic other specify | 30 (0.35) | 501 (15.62) |
| Malignancy: primary other specify | 11 (0.13) | 122 (3.80) |
| Immunosuppressive drug-related | 4 (0.05) | 3 (0.09) |
| Others | 569 (6.68) | 172 (5.36) |
| Unknown | 1,026 (12.05) | 350 (10.91) |
